# Accurate MS-Based Diagnostic Amyloid Typing Using Endogenously Normalized Protein Intensities in Formalin-Fixed Paraffin-Embedded Tissue

**DOI:** 10.1016/j.mcpro.2025.101040

**Published:** 2025-07-21

**Authors:** Vanessa Hollfoth, Arslan Ali, Eyyub Bag, Philip Riemenschneider, Sven Mattern, Julia Luibrand, Mohamed Ali Jarboui, Kerstin Singer, Benjamin Goeppert, Mirita Franz-Wachtel, Martina Sauter, Shabnam Asadikomeleh, Tobias Feilen, Christian Hentschker, Silvia Ribback, Elke Hammer, Karsten Boldt, Frank Dombrowski, Oliver Schilling, Boris Macek, Marius Ueffing, Karin Klingel, Stephan Singer

**Affiliations:** 1 Department of Pathology and Neuropathology, University Hospital Tübingen, Tübingen, Germany; 2 Dr Senckenberg Institutes of Pathology and Human Genetics, University Medicine Frankfurt, Frankfurt, Germany; 3 Core Facility for Medical Proteomics, University Hospital Tübingen, Tübingen, Germany; 4 Institute of Pathology and Neuropathology, RKH Hospital Ludwigsburg, Ludwigsburg, Germany and Institute of Tissue Medicine and Pathology (ITMP), University of Bern, Bern, Switzerland; 5 Proteome Center Tübingen, University of Tübingen, Tübingen, Germany; 6 Institute of Pathology, University Medicine Greifswald, Greifswald, Germany; 7 Institute of Pathology, University Hospital Freiburg, Freiburg, Germany; 8 Interfaculty Institute for Genetics and Functional Genomics, University Medicine Greifswald, Greifswald, Germany; 9 German Centre of Cardiovascular Research (DZHK), Partner Site Greifswald, Germany; 10 Institute for Ophthalmic Research, Center for Ophthalmology, University of Tübingen, Tübingen, Germany

**Keywords:** amyloidosis, diagnostics, FFPE, mass spectrometry, proteomics

## Abstract

Amyloidoses are a group of diseases characterized by the pathological deposition of non-degradable misfolded protein fibrils. These include plasma cell neoplasms, chronic inflammatory conditions, and age-related disorders, among others. Precise identification of the fibril-forming, and thereby amyloidosis-type defining protein is crucial for prognosis and correct therapeutic intervention. While immunohistochemistry (IHC) is widely used for amyloid typing, it requires extensive interpretation expertise and can be limited by inconclusive staining results. Thus, mass spectrometry (MS), if available, has been proposed as the preferred method for amyloid typing by international specialized centers (United States and United Kingdom) using primarily spectral counts for quantification. Here, we introduce an alternative method of relative quantification to further enhance the accuracy and reliability of proteomic amyloid typing. We analyzed 62 formalin-fixed, paraffin-embedded (FFPE) tissue samples, primarily endomyocardial biopsies, using liquid chromatography-tandem mass spectrometry (LC-MS/MS) and employed internal normalization of iBAQ values of amyloid-related proteins relative to serum amyloid P component (APCS) for amyloidosis typing. The APCS method demonstrated robust performance across multiple LC-MS/MS platforms and achieved complete concordance with clear cut IHC typed amyloidosis cases. More importantly, it resolved unclear amyloid cases with inconclusive staining results. Additionally, for samples without a distinct fibril-forming protein identified in the standard procedure, *de novo* sequencing uncovered immunoglobulin light chain components, enabling the diagnosis of rare AL-amyloidosis subtypes. Finally, we established machine learning approach (XGBoost) achieving 94% accuracy by using ∼160 amyloid-related proteins as input variables. In summary, the iBAQ APCS normalization method extended by *de novo* sequencing allows robust, accurate, and reliable diagnostic amyloid typing, and can be complemented by an AI-based classification. Careful reviewing of each histological sample and the clinical context, nevertheless, remains indispensable for accurate interpretation.

## Highlights

• Internal normalization achieves robust amyloid typing in routine diagnostic setting.
• Reproducible diagnostic results across various LC-MS/MS set-ups.
• *De novo* sequencing enables diagnosis of rare AL amyloidosis subtypes.
• Machine learning (XGBoost) achieves 94% accuracy in amyloidosis typing.

## Graphical Abstract

**Figure:**
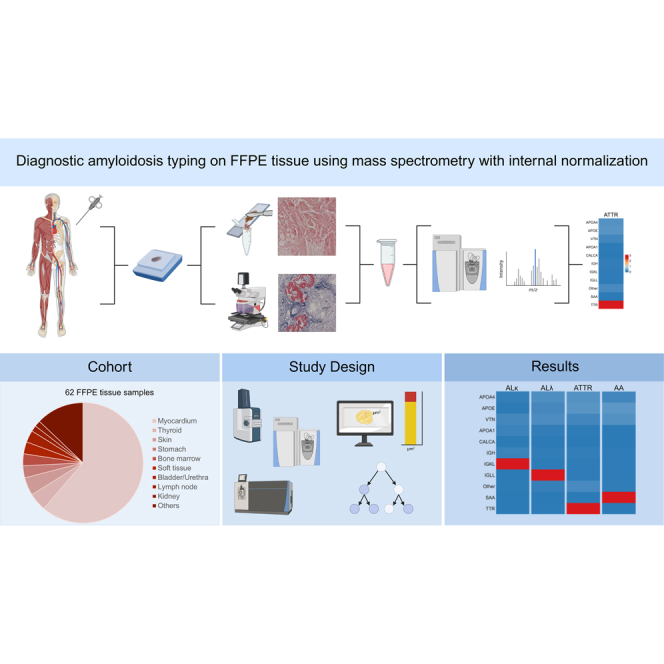

## In Brief

Amyloidoses are diseases caused by misfolded protein fibril deposition. Identifying the fibril-forming protein is crucial for prognosis and treatment. While immunohistochemistry is widely used, mass spectrometry is rising as the diagnostic method of choice. We introduce an internal normalization-based method for amyloid typing, achieving high accuracy across LC-MS/MS platforms, even with low amounts of amyloid. Combined with *de novo* sequencing and machine learning (94% accuracy), this approach enhances amyloid diagnosis.

The term “amyloidosis” defines a disease group characterized by the deposition of misfolded proteins in the interstitium of various tissues through the formation of insoluble fibrils (1, 2). These deposits damage the tissue structure and impair cellular or organ function with varying degrees of severity and progression, depending on the amyloidosis type. To date, over 40 different types have been described, with different underlying causes and preferentially affected organs (3). Systemic amyloidoses most commonly arise as potentially life-threatening complications of hematological diseases (e.g., plasma cell neoplasms leading to light chain amyloidosis (AL)), age-related conditions (transthyretin amyloidosis (ATTR)) or chronic inflammation (serum amyloid A amyloidosis (AA), e.g., in rheumatoid arthritis). Given the wide variety of underlying conditions, amyloidosis diagnosis and typing are critically important for prognosis and therapeutic decision-making (4, 5).

The gold standard for confirming amyloidosis remains the histochemical detection in a tissue sample using Congo Red (CR) staining (6). With this method, amyloid deposits appear red by conventional and mostly exhibit a characteristic apple-green birefringence by polarized light microscopy. In contrast, correct typing by immunohistochemical staining (IHC) is significantly more challenging, as it depends heavily on precise staining procedures, high-quality antibodies, and substantial expertise in interpreting the results. Furthermore, commonly used antibodies typically target only the most frequent amyloidosis types bearing the risk of missing rare types.

The strength of mass spectrometry (MS)-based amyloidosis typing is reflected by the pioneering and successful use of this technology at renowned international amyloidosis centers (e.g. the United Kingdom National Amyloidosis Center, London or the Mayo Clinic, Rochester, United States) for many years. Vrana *et al.* demonstrated 98% to 100% sensitivity and specificity in amyloidosis typing using laser microdissection tandem mass spectrometry (6, 7, 8, 9). In another study from the Mayo Clinic, analyzing amyloidosis samples over an 11-year period, firmly established proteome-based amyloid typing as the method of choice for optimal patient care (10). Accordingly, a variety of publications and consensus statements from leading professional societies (e.g. International Society of Amyloidosis (ISA) and Swiss Amyloidosis Network (SAN)) also emphasize mass spectrometry as the preferred method in amyloidosis typing (2, 4, 6, 11, 12, 13, 14, 15) with its accessibility remaining a notable limitation. Given the broad consensus on the advantages of MS-based amyloidosis typing, the challenge lies in developing a standardized, platform-independent approach to proteomic data acquisition and analysis that simplifies the process, enhances reliability, and facilitates its broader implementation. The previously reported method by Brambilla *et al.* employed a diagnostic cutoff of 70% ɑ-value calculated from spectral counts (16). However, the ɑ-value was calculated using four major subtype-specific proteins only, and in several cases, the diagnostic cutoff was not met (17). Similarly, Vrana *et al.* suggested using the amyloid-associated protein that has the highest spectral count as an indication of the subtype (8). Once a widely used method in quantitative proteomics, spectral counts have seen declining use in the last years as more advanced and accurate techniques have become available.

Here, we propose to use intensity-based absolute quantification (iBAQ) intensities internally normalized to the abundance of the serum amyloid P component (APCS) with the resulting highest peak defining the amyloidosis type. APCS, being universally present in amyloid deposits (18), serves as an ideal “housekeeping” protein for normalization purposes. Furthermore, iBAQ intensities are suggested to correlate with the absolute quantities of proteins particularly facilitating accurate comparisons of protein abundances within a single sample (19). This characteristic is well-suited to the typical diagnostic scenario, where analyses are performed on individual patient samples. We show that this approach performs reliably independent of the LC-MS/MS platform being used and that it is also compatible with cardiac specimens, where the whole section of the biopsy was used for MS-analysis (whole-dissected samples).

## Experimental Procedures

### Experimental Design and Statistical Rationale

This study presents a sample preparation and data analysis workflow to achieve robust amyloidosis typing based on FFPE-tissue proteomics being performed on 62 samples. The key element of this approach is to examine each sample individually, recapitulating the diagnostic situation. Upon LC-MS/MS internal normalization of iBAQ values of amyloid-related proteins relative to serum amyloid P component (APCS) was conducted. By this approach the resulting highest relative abundance of a particular protein (or several proteins in mixed-type amyloidosis) indicated the respective amyloid type. Therefore, no statistical comparisons between any groups of amyloidosis types were required.

For classification using the machine learning algorithm XGBoost, the dataset (including all amyloidosis samples with three or more cases per group) was randomly split into 30 training samples and 18 testing samples. A detailed description of the workflow along with the results is provided in the following sections.

### Patient Cohort

The collection of 62 FFPE-tissue samples used in this study consisted of a variety of tissue samples with suspected diagnosis of amyloidosis obtained for histopathological routine diagnostics between 2000 and 2025. Samples were retrieved from the archives of the Institutes of Pathology at the University Hospital in Tübingen and University Medicine in Greifswald, Germany. Histological and immunohistochemical evaluation of every sample was performed by experienced pathologists (K. K., S. S., J. L., S. M., K. S., S. R., F. D.). Patient characteristics are summarized in Supplemental Table S1.

### Ethics

The study was approved by the local ethic committee of the medical faculty of the Eberhard-Karls-University Tübingen in the context of an amendment to the existing vote 411/2021BO2. Written informed consent from patients was acquired and the study conformed to the principles set out in the WMA Declaration of Helsinki and the Department of Health and Human Services Belmont Report.

### Histological Staining and Immunohistochemistry

For CR staining, FFPE specimens were cut into 7-μm-thick sections and mounted on TOMO adhesive glass slides (Matsunami Glass Ind., Ltd). The slides were deparaffinized by incubation in Xylene for 15 min and then 2x EtOH 100%, 2x EtOH 96%, 2x EtOH 70% for 1 min each, before washing with distilled water. Staining is performed using nuclear staining (according to Mayers) for 10 min, followed by washing with tap water for 10 min. Then, Congo staining solutions I and II (according to Puchtler) are applied consecutively for 10 and 60 min, respectively, followed by washing 6-12 times in distilled water. The slides are then incubated 1x in EtOH 100% and 2x in Xylene for 1 min each before mounting.

For immunohistochemistry we used heat-induced epitope retrieval. After incubation with the antibodies (Transthyretin (Dako, Code No. A0002), Amyloid A (Dako, Code No. M0759), Kappa Light Chains (Dako, Code No. A0191), Kappa Light Chain (Novus, NBP2-15191), Lambda Light Chains (Dako, Code No. A0193) and Lambda Light Chain (Leica, NCL-L-LAM-578)) we used ZytoChem Plus (HRP) Polymer Kit (Zytomed) for detection followed by HistoGreen (Linaris) as substrate.

### Processing of FFPE Samples for Proteomic Analyses

**Fig. 1:**
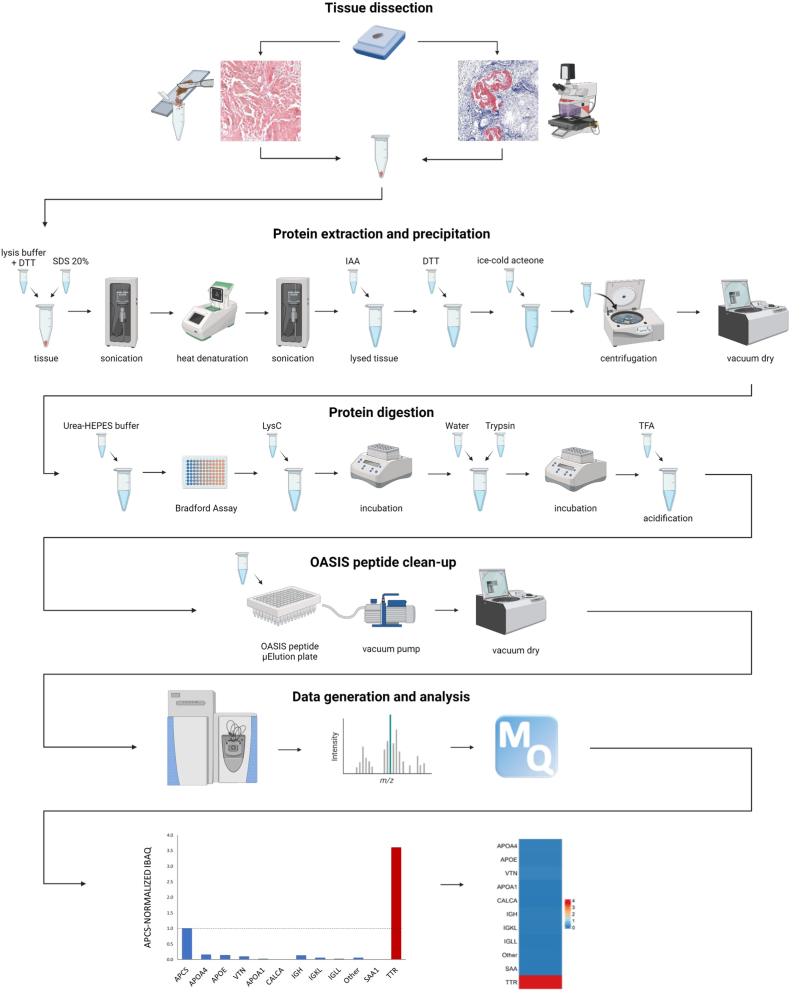
**Workflow—Preparation of amyloidosis samples for LC-MS/MS and data analysis Congo Red (CR) staining of FFPE tissue samples and subsequent macro- or microdissection, depending on the distribution of amyloid.** Proteins were extracted, precipitated and digested, resulting peptides were cleaned up, dried and transferred to different proteomics core facilities for liquid chromatography-tandem mass spectrometry (LC-MS/MS) measurements. Data analysis (*bottom*) includes internal normalization of iBAQ values to APCS for each sample and assignment of amyloidosis type based on the highest peak of selected amyloid-related proteins. Created in BioRender.

The approach suggested for mass spectrometry-based amyloid typing consists of three major steps: Tissue dissection, sample preparation, and measurement with subsequent data analysis (Fig. 1). Tissue dissection is performed either via macrodissection or laser-capture microdissection based on the size and distribution of amyloid tissue. In the second step, the tissue undergoes protein extraction, precipitation, digestion, and clean-up using a previously published protocol suitable for FFPE tissue (20) based on the work of Buczak *et al.* (21). Digested peptides are then subjected to LC-MS/MS analysis employing DDA analysis methods followed by MaxQuant-based protein identification.

#### Macrodissection

FFPE specimen were cut in 5-μm-thick sections and mounted on TOMO adhesive glass slides (Matsunami Glass Ind., Ltd). For each case an additional 7-μm section was prepared and stained with CR. On this slide the area for macrodissection was marked by an experienced pathologist. Then the unstained slides were deparaffinized by incubation in 2x Xylene for 2 min and then 2x EtOH 100% for 2 min before drying.

Afterwards, macrodissection of the marked areas was performed with a scalpel (No. 15, Feather) using the CR staining as a reference. To avoid powder forming, small amounts of ultra-pure water (New England Biolabs) were added to keep the blade moist. The tissue was collected in 0.5 ml tubes (Eppendorf).

#### Microdissection

FFPE specimen were cut in 5 μm thick sections and mounted on PEN membrane slides (Targeted Biosciences, Inc.) and stained with CR. For each case an additional 7 μm section was prepared on a conventional slide and stained with CR and covered with a cover slip. On this slide the area for microdissection was marked by an experienced pathologist. Then, the uncovered membrane slides were deparaffinized by incubation in 2x Xylene for 2 min and then 2x EtOH 100% for 2 min before drying. Afterwards, microdissection of the marked areas was performed with a Laser Capture Microdissection Microscope (Targeted Biosciences, Inc) using the CR staining as a reference. The tissue was collected in 0.5 ml tubes (Eppendorf).

#### Protein Extraction

100 μl DTT-containing extraction buffer (4% SDS, 80 mM DTT in 1 M Tris pH 8.0) was added to each tissue sample. The samples were then sonicated for 15 cycles (20 °C, 1 min ON; 30 s. OFF, setting: HIGH) using a Bioruptor Plus (Diagenode) followed by heating to 99 °C for 1 h in a PCR machine. These two steps were repeated once, followed by another sonication step. Iodoacetamide, (IAA, Sigma/Merck) was added to a final concentration of 15 mM and the samples were incubated in the dark for 30 min to allow alkylation of free cysteines. The reaction was quenched by the addition of DTT (Dithiothreitol, Sigma/Merck) to a final concentration of 10 mM before transferring samples to a fresh 1.5 ml reaction tube (Eppendorf).

#### Protein Precipitation

For protein precipitation 8x the volume (952 μl) of 100% ice-cold acetone (Biosolve) was added to each sample and then stored at −20 °C overnight. Centrifugation of the samples was performed at 21,000*g* and 4 °C for 30 min. Acetone was removed, and the pellets were washed with 500 μl ice-cold 80% acetone and subsequently centrifuged at 4 °C for 10 min. The washing step was repeated, followed by 2 min of centrifugation under the same conditions. The supernatant was discarded, and the samples were completely dried for 15 min using a centrifugal vacuum concentrator (Concentrator Plus, Eppendorf).

#### Protein Digestion

Dried samples were resuspended in 46 μl of 3 M urea in 100 mM 4-(2-hydroxyethyl)-1 piperazineethanesulfonic acid (HEPES, both from Carl Roth GmbH) by using the Bioruptor Plus for 5 cycles (60 s ON, 30 s OFF, setting HIGH) until the pellet completely dissolved. The protein yield was estimated via Bradford assay (Thermo Fisher Scientific) according to the manufacturer’s specifications and absorbance was measured using an iMarkTM Microplate Absorbance Reader (Bio-Rad, Hercules).

For each sample up to 5 μg of protein were transferred into a fresh 1.5 ml Eppendorf tube and filled up to 40 μl with 3 M urea in 100 mM HEPES. 0.5 μl of LysC (0.5 μg/μl, FUJIFILM Wako pure chemicals corporation) were added and the samples were incubated for 4 h at 37 °C and 600 rpm using the Thermomixer comfort (Eppendorf). Afterward, 40 μl ultra-pure water was added to each sample, followed by 0.5 μl of Trypsin (0.5 μg/μl, Promega), and incubation at 37 °C and 600 rpm for 16 h. In samples that yielded less than 5 μg of protein, amounts of LysC and Trypsin were adjusted accordingly.

#### Peptide Clean-Up

After acidification with 7.4 μl of 10% trifluoroacetic acid (Biosolve, Dieuze, France), the samples were desalted using a vacuum assisted solid phase extraction (Waters OASIS HLB μElution plate 30 μm; Waters) according to the manufacturer’s instructions. The samples were collected in 0.2 ml tubes (Ratiolab), dried in the Concentrator Plus (45 °C, AQ, 45 min), and stored at −20 °C until mass spectrometric measurement.

### Liquid Chromatography and Tandem Mass Spectrometry

LC-MS/MS was mainly performed at the Core Facility for Medical Proteomics Tübingen. Here, 250 ng of peptides were loaded onto an Ultimate 3000 LC system coupled to a QExactive Plus mass spectrometer (all Thermo Electron).

Peptides were separated using a linear gradient from 2% to 30% of buffer B (80% acetonitrile and 0.08% formic acid in HPLC-grade water) and buffer A (2% acetonitrile and 0.1% formic acid in HPLC-grade water) at a flow rate of 300 nl per min for 39 min. The remaining peptides were eluted by changing the gradient from 30% to 95% buffer B in 1 min. Followed by maintaining 2% of buffer B for 20 min, the total runtime was 70 min. Data were acquired in DDA mode with an MS1 mass range of 335 to 1500 m/z with Orbitrap resolution set at 70K, AGC target for MS1 was set to 3 × 10E6 with a maximum injection time of 100 ms. The precursor ion selection was done by setting TopN at 10. The MS2 scan range was 200 to 2000 m/z with Orbitrap resolution set at 17.5K, the AGC target to 1 × 10E5, and the maximum injection time to 200 ms. Both MS1 and MS2 data were acquired in centroid mode.

For validation, a subset of case samples was remeasured at the Core Facility for Medical Proteomics Tübingen (Ultimate 3000 + Orbitrap Fusion), as well as the Proteome Center Tübingen (Easy-nLC + QExactive-HF), the Department of Functional Genomics of the University Medicine Greifswald (nano-Acquity + LTQ-Velos/Ultimate 3000 + QExactive plus) and the Institute of Pathology of the University Hospital Freiburg (Evosep one + TimsTOF). Detailed information on the LC and MS machines and settings used for each sample are stated in Supplemental Table S2.

### Data Analysis

All raw files were processed using the MaxQuant Software (Version 2.1.0.0) and searched against the human subset of SwissProt database + isoforms (42.383 entries, downloaded 2022/08/01), without using match-between-runs option. The following search parameters were set: 1) Requirement of full tryptic specificity (cleavage after lysine or arginine residues, unless followed by proline); 2) Only two missed cleavages were allowed; 3) Carbamidomethylation (C) was used as fixed modification; 4) Variable modifications were oxidation (M) and acetylation (protein N-term); 5) 4.5 ppm for precursor ions and 20 ppm for MS/MS were set for mass tolerance for samples measured on QExactive (20 ppm precursor and 0.5 Da MSMS (Fusion), 20 ppm precursor and 40 ppm MSMS (TimsTOF), ppm precursor and 10 ppm and 0.6 Da MSMS (LTQ Velos)). The reversed sequences of the target database served as a decoy database. The false discovery rate was set to 1% at both peptide and protein level using target-decoy strategy.

The protein identification results from the search output were evaluated for the number of unique peptides identified of four amyloid signature proteins (serum amyloid P component (APCS), apolipoprotein E (APOE), apolipoprotein A-IV (APOA4), and vitronectin (VTN)). Amyloid signature protein (ASP)-test is considered positive if three out of four proteins could be definitely identified by more than 2 unique peptides. Amyloidosis subtyping is conducted by plotting the APCS-normalized iBAQ intensities of all major amyloid proteins (3, 22). The list was extended by proteins that are known to be similar to the published proteins (especially a variety of variable immunoglobulin chains, Supplemental Table S3). The protein representing the highest intensity on the plot (except for APCS) denotes the subtype of amyloidosis and is visualized in a bar diagram or heatmap. Analysis of the amyloid percentage area in whole-dissected samples was performed using the pixel classifying function of QuPath-0.5.1 (23).

### De novo Sequencing

Unidentified spectra with highest precursor ion intensities (Top 100) were exported to a new mgf file and *de novo* sequencing was performed with Novor (24) via DeNovoGUI 1.16.8 (CompOmix) (25). Modifications and allowed mass tolerances were identical to the database search approach. Hits with a Novor score above 60 were considered for a protein BLAST approach. Protein BLAST for the sequenced peptides was conducted with the NCBI BLASTp algorithm (BLAST+ 2.15.0) with default settings against non-redundant protein sequences narrowed down to humans (taxid:9606). Alignments were chosen according to the Expect (E)-values (with lowest among hits), the query coverage, and if the homologous proteins were related to amyloidosis. Here, E-values corresponds to the number of hits that can occur by chance (https://www.nlm.nih.gov/ncbi/workshops/2023-08_BLAST_evol/e_value.html). With this information, a modified Swissprot database was created to conduct MaxQuant searching for further analyses and protein quantification.

### XGBoost

Extreme Gradient Boosting (XGBoost) is a supervised machine learning method which combines bagging and boosting methods for better classification performance. It combines many individual trees, which are generated iteratively on random subsets of training samples and features while trying to optimize the performance in each iteration. An implementation for this method with many state-of-the-art features regarding scaling, optimization, and customization is provided in the open-source library XGBoost. [https://dl.acm.org/doi/10.1145/2939672.2939785]

We used the xgboost (Version 1.7.8.1) and caret (Version 6.09–94) packages (https://cran.r-project.org/web/packages/caret/citation.html) in R (Version 4.3.3) for training and hyperparameter optimization of our XGBoost classifier. Our dataset of all samples with three or more cases per group was randomly split into 30 training samples and 18 testing samples. The hyperparameters were tuned with a repeated cross-validation strategy on random subsets of our training data. The parameters yielding the best accuracy on our data were chosen for our classifier. In a similar fashion, we additionally trained a support vector machine and a random forest classifier, using the kernlab (Version 0.9–32) and ranger (Version 0.16.0) packages, respectively, to compare the resulting performances.

## Results

### Internal Normalization of iBAQ Values to APCS for Tissue-Based Amyloidosis Typing

The proposed workflow using the iBAQ APCS internal normalization method consists of five major steps: 1) tissue dissection of CR positive amyloid deposits (either through macrodissection or laser capture microdissection (LMD)); 2) protein extraction and precipitation; 3) protein digestion; 4) peptide clean-up; and 5) data generation and analysis, as illustrated in Figure 1. For relative quantification, iBAQ values of amyloid-related proteins were internally normalized to the amyloid signature protein APCS. Amyloidosis-type assignment was based on the highest relative intensity of amyloid-related proteins, which is visually represented in red within the bar plot or heatmap. This approach is exemplified by using a previously by IHC diagnosed cardiac ATTR case, which resulted in the expected diagnosis of ATTR (Fig. 1). Besides APCS, the proteins APOA4, APOE, and VTN are recognized as amyloid signature proteins (2, 26). These proteins are present in varying amounts across all or most amyloidosis types; however, with the exception of APOA4 in rare cases (as demonstrated below), they are not considered causative amyloidogenic proteins. As such, these proteins serve as additional quality control markers, complementing the initial histological and histochemical quality checks of each amyloidosis sample, and are shown in all following heat maps/bar diagrams. For proteins associated with immunoglobulin heavy chains (IGH) and kappa (IGKL) and lambda (IGLL) light chains, all constant and variable regions detected for each type are grouped and summarized for a more concise presentation as IGH, IGKL, and IGLL, respectively. Each heatmap highlights the highest relative value for each group, as exemplified in Supplemental Figure S1. The “Other” category included additional amyloidosis-related proteins identified through previous proteomic analyses (2, 3, 22, 26) and were further expanded by the findings of this study. All protein groupings and group members are detailed in Supplemental Table S3. Results from MaxQuant search are provided as Supplemental Table S6 and publicly available as detailed in the Data Availability statement.

### Internal iBAQ APCS Normalization is Applicable to Whole-Dissected Endomyocardial Biopsies Even With Low Amyloid Content

**Fig. 2:**
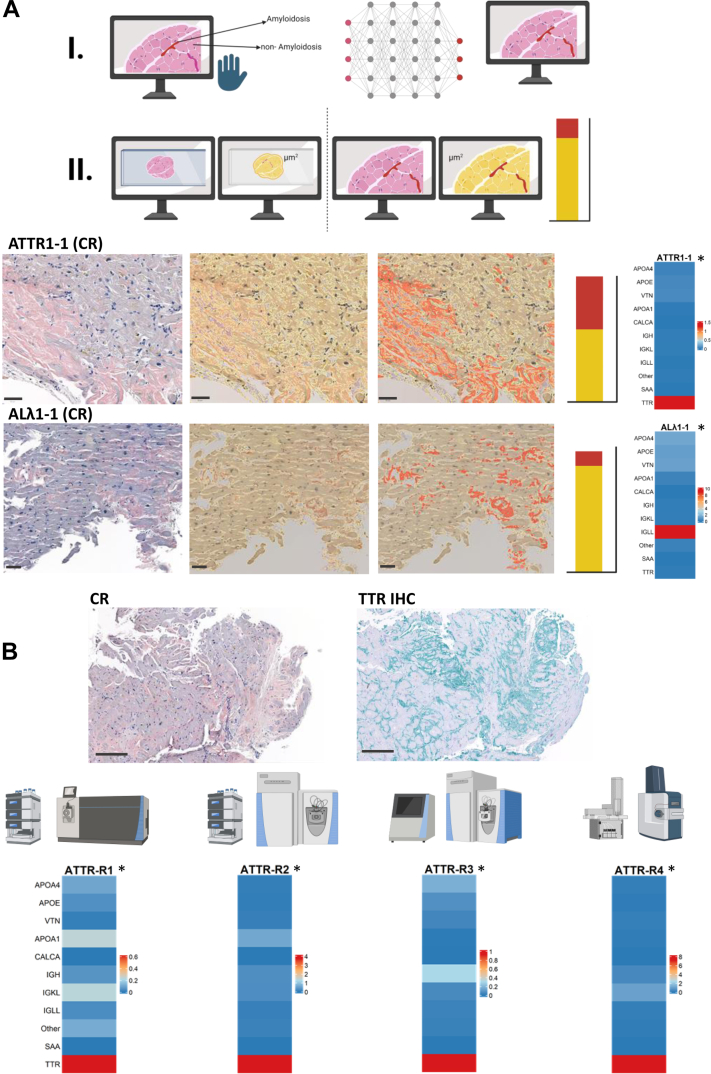
**Method validation using various machines and different percentages of amyloid.***A*, determination of amyloid contents in whole-dissected myocardial samples using QuPath software. Scheme illustrates training of the software with tissue- and amyloidosis-positive and negative areas (I.) and afterwards application to whole-dissected tissue slides (II.). Histological scans (scale bar 50 μm) show two amyloidosis samples in CR staining, whole tissue selection and determination of amyloid percentage by QuPath software (from *left* to *right*). Example 1 (ATTR1-1, *upper panel*) yielded 40% amyloid, whereas in Example 2 (ALλ1-1, *bottom panel*) 9% of amyloid were sufficient for amyloid typing, as shown in the heatmap (*right*). *B*, cross validation of a clear-cut myocardial ATTR sample, diagnosed by CR staining and typed by IHC (*upper panel*, scale bar 200 μm), was measured proteomically across several machines (Orbitrap Fusion, QExactive, QExactive-HF and TimsTOF) at different proteomics facilities in Tuebingen and Freiburg. Amyloid typing was successful, irrespective of the machines tested (*bottom panel*). Whole-dissected samples are indicated with an asterisk (∗). Created in BioRender.

**Fig. 3:**
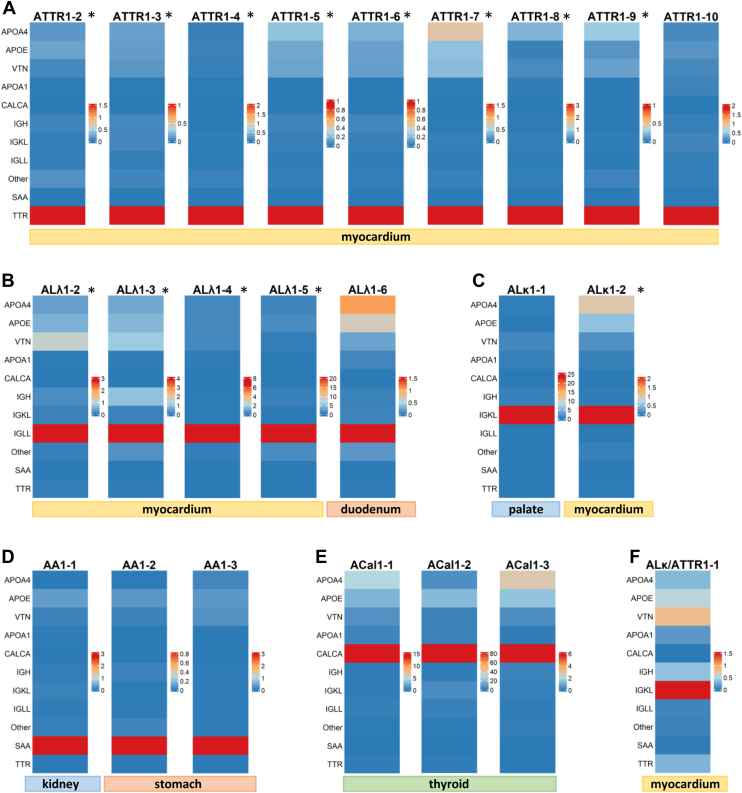
**Standard amyloidosis cases consistent with IHC or clear-cut based on clinical background.** Heatmaps illustrating amyloid typing of clear-cut amyloidosis samples, where proteomics results were consistent with IHC staining (ATTR (*A*), Alλ (*B*), Alκ (*C*), AA (*D*)) or determination of amyloidosis type was consistent with the histological context (ACal (*E*)). Additionally, a mixed/double amyloidosis case was confirmed by LC-MS/MS to include both kappa light chains and TTR (*F*). Whole-dissected samples are indicated with an asterisk (∗).

**Fig. 4:**
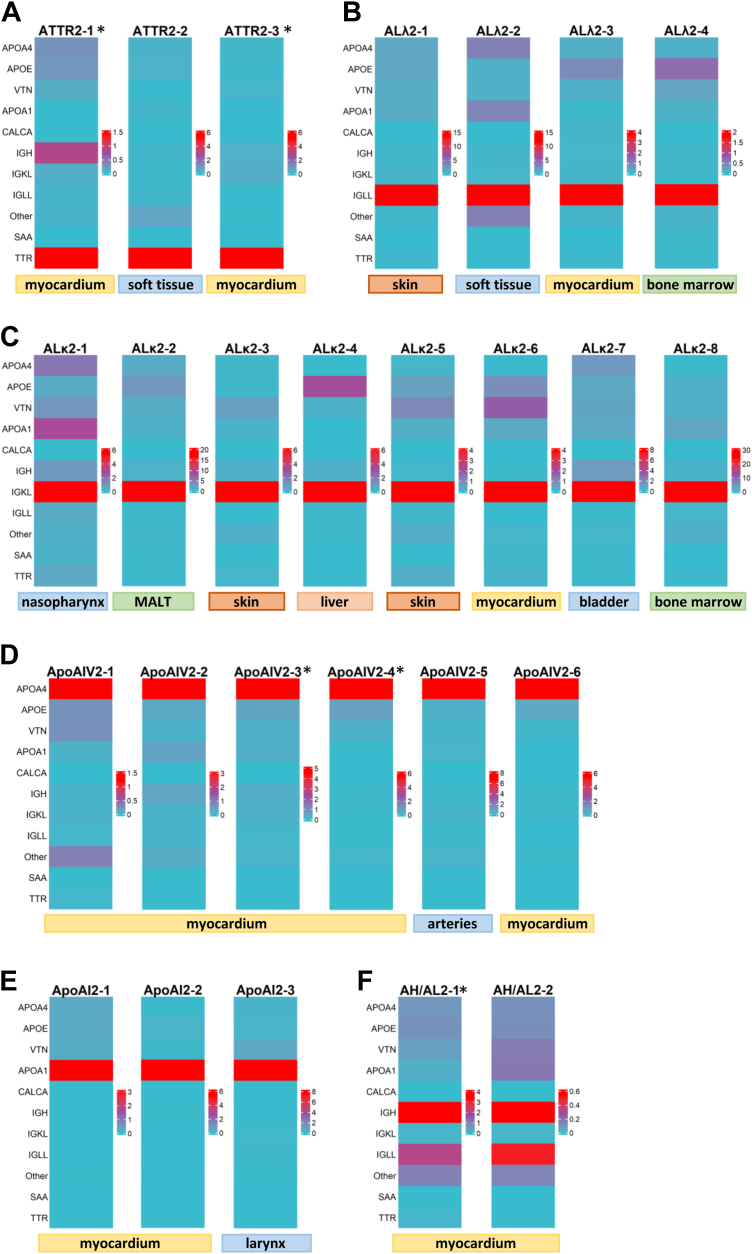
**Challenging cases with inconclusive or no available IHC.** Heatmaps illustrating amyloid typing of challenging samples, including ATTR (*A*), ALλ (*B*) and ALκ (*C*) where IHC was inconclusive. *D* and *E* show rare amyloid types ApoAI and ApoAIV with no available IHC. In mixed AH/AL amyloidosis (*F*) only light chains are detected by IHC, whereas heavy chain deposits are often missed. Whole-dissected samples are indicated with an asterisk (∗).

When typing cardiac amyloidosis in endomyocardial biopsies a frequent diagnostic challenge are amyloid deposits being finely branched and diffusely distributed between the cardiomyocytes, rendering them largely inaccessible to LMD. To address this, we tested whether our approach could also be applied to whole-dissected endomyocardial biopsies. In the initial step, we used the QuPath software to estimate the percentage of the amyloid area relative to the total biopsy area for a given sample. Figure 2*A* illustrates histological scans of two exemplary whole-dissected myocardial samples (ATTR1-1 and ALλ1-1), where amyloid content was determined by QuPath being 40% and 9%, respectively. The tissue scans (left panel) were evaluated using a pixel classifier that applies a pixel threshold to distinguish tissue from background, thereby identifying the entire tissue area (middle panel, highlighted in transparent yellow). The amyloid area (right panel, highlighted in transparent red) was estimated by QuPath, which was trained before using slides where amyloid-positive and -negative regions were annotated by an experienced pathologist. The amyloid typing results are visualized in heatmaps, providing a definitive diagnosis consistent with the IHC staining (ATTR1-1 and ALλ1-1, respectively, Fig. 2*A*). Additional successfully typed amyloidosis cases based on whole-dissected endomyocardial biopsy are shown below as part of Figures 3, 4, and Supplemental Figure S2 (marked with an asterisk (∗)) with the lowest amyloid content being 1.7% in an ATTR case. For a list of all whole dissected samples and estimations by QuPath see Supplemental Table S4.

To validate the robustness of our approach across different analytical platforms, we analyzed MS data from a representative whole dissected ATTR sample (ATTR2-3) using four major LC-MS/MS instrument combinations hosted at different Proteomic Core Facilities: ATTR-**R1** (Ultimate 3000 LC + Orbitrap Fusion Tribrid), ATTR-**R2** (Thermo-nanoLC + QExactive), ATTR-**R3** (Easy-nLC + QExactive-HF), and ATTR-**R4** (Evosep one + TimsTOF), as shown in Figure 2*B*. The results consistently demonstrate accurate amyloidosis typing of each replicate as ATTR across all platforms, in full concordance with the IHC staining result (Fig. 2*B*, upper panel). These data confirm the platform-independent reliability of the proposed method.

### Confirmation of Clear-Cut IHC-Typed Amyloidosis Samples

Having conducted several validation steps including different LC-MS/MS instrumentation and different sample formats, as outlined above, we applied the approach to 23 representative amyloidosis cases. These covered major amyloidosis types (ATTR (n = 9), ALλ (n = 5), ALκ (n = 2), AA (n = 3)) with an unequivocal diagnosis based on IHC (also including a mixed amyloidosis (ALκ/ATTR, n = 1)) or the histological context (calcitonin amyloidosis in medullary thyroid carcinoma (ACal, n = 3)). The samples were primarily of cardiac origin (n = 15) but also included other localizations such as thyroid (n = 3), kidney (n = 1), stomach (n = 2), duodenum (n = 1), and palate (n = 1), as indicated below each heatmap in Figure 3. As shown in Figure 3, *A*–*E*, all of these amyloidosis types could be clearly confirmed with varying relative highest abundances of TTR (ATTR1-2 to ATTR1-10; Fig. 3*A*), IGLL (ALλ1-2 to ALλ1-6; Fig. 3*B*), IGKL (ALκ1-1 to ALκ1-2; Fig. 3*C*), SAA (AA1-1 to AA1-3, Fig. 3*D*), and CALCA (ACal1-1 to ACal1-3, Fig. 3*E*). Additionally, the mixed amyloidosis sample from the heart, which showed IHC positivity for both TTR and IGKL (in different areas and amounts within the sample), was confirmed as ALκ/ATTR1-1. While the abundance of TTR was lower than IGKL (since TTR represented the minor component in this mixed amyloidosis sample), it was still notably higher than in non-ATTR cases, where TTR is typically not detected in significant amounts (Fig. 3*F*).

Moreover, we analyzed six additional myocardial samples with ALλ (n = 5) and ATTR (n = 1) showing unequivocal IHC results, but two or more relatively high abundant proteins without representing truly mixed amyloidosis types (Supplemental Fig. S2*A*). Additional “peaks”, primarily among the amyloid signature proteins APOA4, APOE and VTN have been reported before (2), but should not be misinterpreted as indicative of mixed amyloidosis. APOE and VTN are generally not regarded as causative amyloidogenic proteins, and consequently, there is no recognized APOE or VTN amyloidosis as defined by the latest classification of the ISA (3). While there are ApoAIV amyloidosis cases, these typically feature APOA4 as the sole prominent peak (see below). To the best of our knowledge, true mixed amyloidosis cases involving APOA4 have not been described so far. As opposed to the aforementioned signature proteins being intrinsic components of amyloid deposits, elevated levels of FGA and KRT—except in cases of kidney or corneal amyloidosis—are more likely attributable to contamination from fibrin exudates or adjacent epithelial cells (as confirmed for both of these samples by re-evaluating the dissected area of the tissue). Accordingly, these proteins should be excluded from consideration in amyloidosis typing. Taking these precautions into account, all six additional cases of amyloidosis (CON1-6) were accurately classified according to the IHC results (Supplemental Fig. S2*A*).

These findings indicate full consistency between unequivocal IHC typed amyloidosis samples and proteomic typing using the iBAQ APCS normalization approach. However, they also underscore the critical importance of integrating histological characteristics, anatomical localization, and clinical context when interpreting proteomic results to ensure accurate and reliable amyloid typing.

### Resolving Challenging Amyloidosis Cases With Equivocal IHC Staining

We further applied the proposed approach to 26 challenging cases where IHC-based typing either failed to yield an unequivocal result or suggested the possibility of mixed amyloidosis. While ATTR is usually well recognizable by IHC, the examples shown in Figure 4*A* (ATTR2-1 to ATTR2-3; myocardium (n = 2) and soft tissue (n = 1)) demonstrated cases that also exhibited potentially positive antibody staining for λ or κ light chains. However, the proteomic analyses revealed high intensities exclusively for the TTR protein. With no significant additionally high-abundant proteins from the λ or κ light chain group the diagnosis of a “pure” ATTR could be made and a mixed amyloidosis could be ruled out. Figure 4*B* (ALλ2-1 to ALλ2-4; skin (n = 1), soft tissue (n = 1), myocardium (n = 1) and bone marrow (n = 1)) and 4C (ALκ2-1 to ALκ2-8; nasopharynx (n = 1), MALT (n = 1), skin (n = 2), liver (n = 1), myocardium (n = 1), bladder (n = 1) and bone marrow (n = 1)) depict AL cases with inconclusive IHC staining that were definitively typed as either ALλ or ALκ by LC-MS/MS. Notably, here ALκ cases were more frequent compared to those in Figure 3, indicating that ALκ is more challenging to be detected via IHC compared to ALλ, especially in non-myocardial biopsies, even when utilizing multiple antibodies per subtype.

ApoAI and ApoAIV amyloidosis belong to rare amyloidosis types and contain APOA1 and APOA4 as amyloidogenic proteins, respectively. They are usually not covered by the IHC antibody repertoire in pathology institutes and thereby are particularly susceptible to be overlooked or misdiagnosed. Adding to the diagnostic challenge ApoAI and ApoAIV amyloidosis can mimic common forms of cardiac amyloidosis (27). As demonstrated in Figure 4, *D* and *E*, the implemented workflow successfully identified both subtypes (ApoAIV2-1–ApoAIV2-6; myocardium (n = 5) and arteries (n = 1)) and (ApoAI2-1 - ApoAI2-3; myocardium (n = 2) and larynx (n = 1)) with unequivocal results. All six ApoAIV samples displayed consistently high intensity ratios for the APOA4 protein, while other amyloidosis-relevant protein intensities were low or absent (Fig. 4*D*). Similarly, the APOA1 protein intensity ratio was the highest in samples ApoAI2-1 - ApoAI2-3 (Fig. 4*E*).

Moreover, proteomic analysis enabled the identification of two rare myocardial AL/AH (mixed immunoglobulin light chain/heavy chain) samples (Fig. 4*F*). The heatmap for these samples revealed high intensity ratios for both immunoglobulin light-chain (IGL) and immunoglobulin heavy-chain (IGH) proteins, with the latter not being included in regularly used IHC antibody panels.

As outlined earlier, we were able to additionally type three samples of ALλ (CON7; lymph node (n = 1)), ATTR (CON8; bursa (n = 1)), and ALκ (CON9; myocardium (n = 1)) with unspecific or negative IHC results, but additional high abundance of ApoA4, FGA, and KRT, respectively, without defining the amyloidosis type (Supplemental Fig. S2*B*).

In conclusion, the implemented workflow has proven effective in resolving challenging, complex, and rare amyloidosis subtypes that could not be definitively diagnosed using IHC.

### *De novo* Sequencing is Required for Identifying Rare Subtypes of AL Amyloidosis

**Fig. 5:**
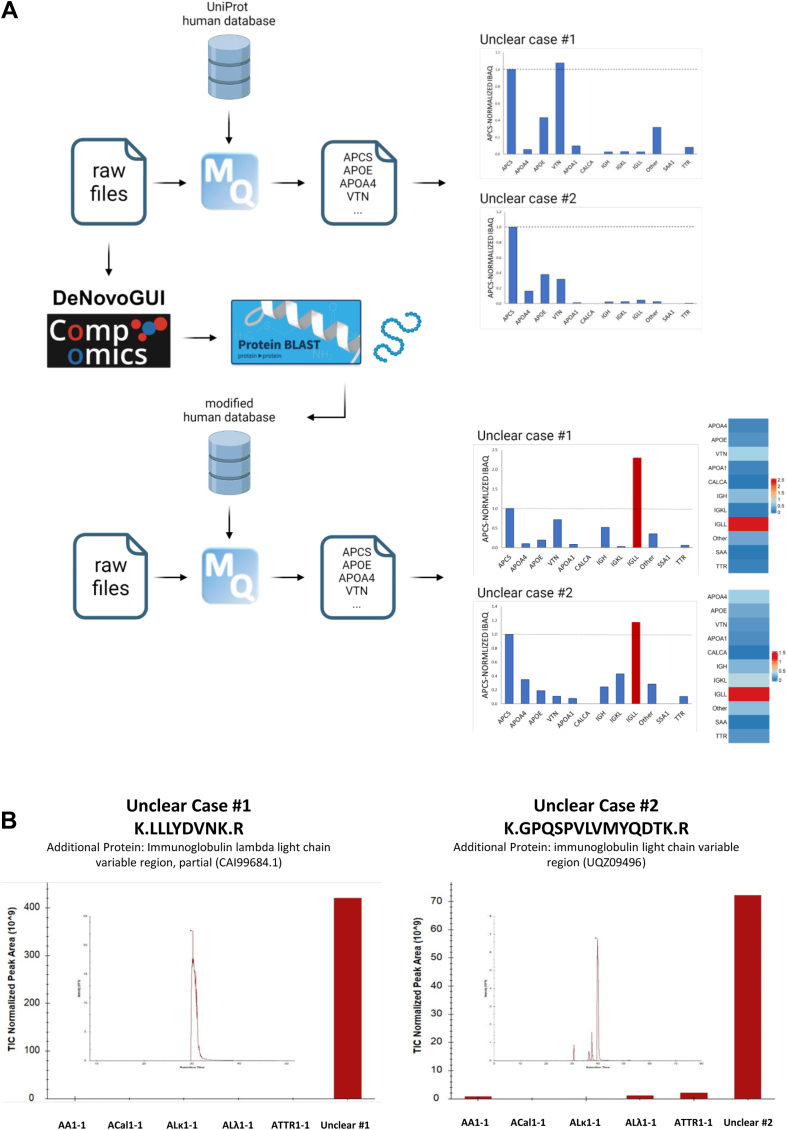
**Tacklingunclear samples where ASP test is positive, however, typing is not achieved.***A*, Bar diagrams and heatmaps of two example cases with high iBAQ intensities of signature amyloid proteins; however, no subtype-specific protein with significantly high intensity was found (A, *upper panel*). Additional search workflow using *de novo* sequencing followed by NCBI Blastp search to retrieve new protein candidates not found in the Swissprot human database. The candidate proteins are added to the human Swissprot fasta database to re-search raw files for estimating iBAQ intensities. The iBAQ intensities for newly added proteins can be used to type unclear samples. The bar diagrams and heatmaps of two example cases where iBAQ intensities of newly found proteins clearly indicating the amyloidosis subtype (A, *bottom panel*). *B*, iBAQ intensities and extracted ion chromatograms of unique peptides of the candidate proteins compared to other amyloidosis subtypes. Created in BioRender.

In rare instances, amyloidosis samples exhibiting positive CR staining with birefringence under polarized light, negative IHC results, and a positive ASP test, may not display a distinctive amyloidosis type-defining peak upon our standard approach. This is exemplified by two cases (Fig. 5*A*, upper panel). Here, additional steps, including *de novo* sequencing, were necessary (Fig. 5*A*, lower panel). Upon subjecting the MS/MS spectra of highest intensities to *de novo* sequencing, we identified peptide sequences such as LLLYNVDK (Unclear Case #1), and GPQSPVLVMYQDTK (Unclear Case #2) (Fig. 5*B*). Subsequent NCBI BLAST searches for these peptide sequences revealed several proteins corresponding to IGL proteins. Proteins with strong E-scores (>60), such as “immunoglobulin lambda light-chain variable region, partial” (CAI99684.1; Unclear Case #1) and “immunoglobulin light chain variable region” (UQZ09496; Unclear Case #2), were identified, downloaded, and incorporated into the used human SwissProt FASTA database (Supplemental Fig. S3). The updated MaxQuant search demonstrated that the iBAQ intensities of these proteins were among the highest in the samples. Bar charts and heatmaps resulting from these data clearly indicated the presence of ALλ amyloidosis (Fig. 5*A*, lower panel). Additionally, extracted ion intensities of these peptides revealed high peak areas specific to these cases, which were absent in other clearly defined amyloidosis samples (Fig. 5*B*). Importantly, for both samples the clinical context aligned with ALλ (case#1 = localized nodular penile amyloidosis, case #2 = clinically suspected AL-amyloidosis).

We conclude that *de novo* sequencing is essential for diagnosing rare AL subtypes that do not present a distinctive peak in the standard iBAQ ASPC normalization procedure.

### A Machine Learning Algorithm (XGBoost) Accurately Classifies 94% of Amyloidosis Cases Using a Curated Set of Amyloidosis-Related Proteins

**Fig. 6:**
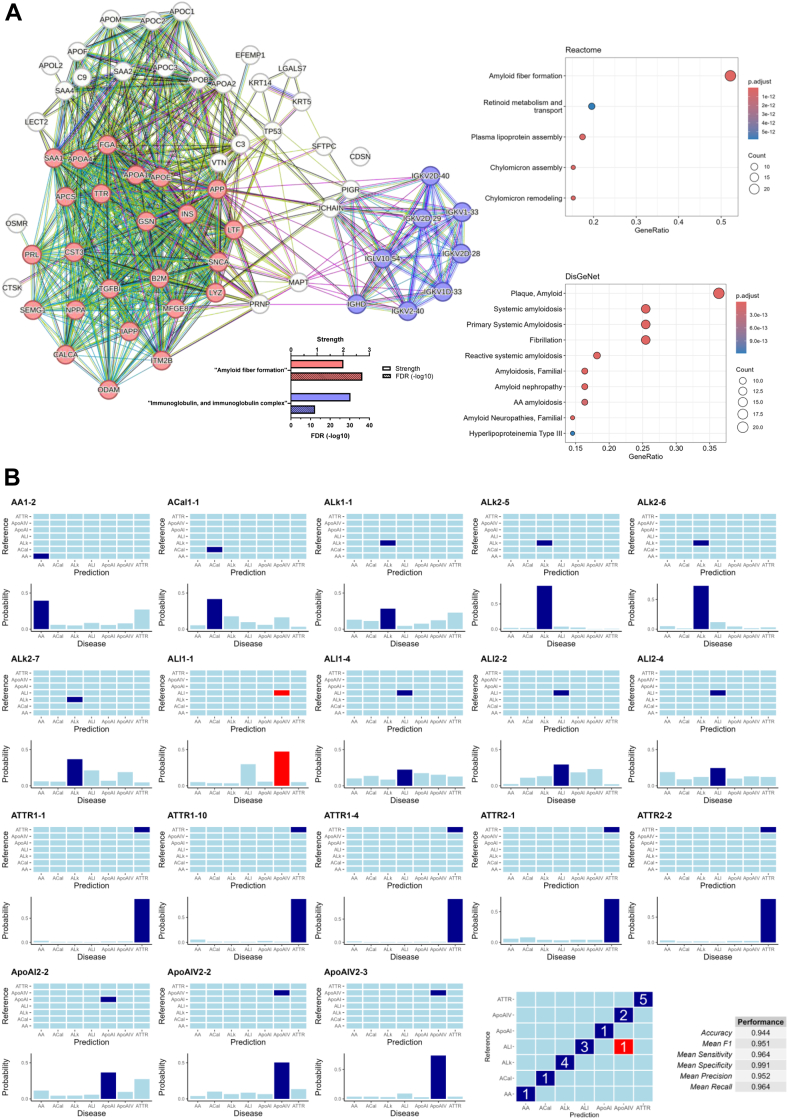
**Enrichment of amyloidosis-related proteins and AI-based classification model using XGBoost.***A*, STRING enrichment analysis of amyloidosis-related proteins (8 out of 107 immunoglobulins were matched by STRING), proteins colored in *red* belong to Reactome enrichment term “Amyloid fiber formation” as displayed on the *right*. *Blue color* indicates STRING enrichment term “Immunoglobulin, and immunoglobulin complex”. Enrichment by the DisGeNet database (*bottom right*) indicates a high correlation of the selected proteins to amyloidosis. *B*, AI-based classification via XGBoost, trained on a random split of all amyloidosis types with three or more cases per group. The resulting classifications and their class probabilities are illustrated for each sample in the testing set. General performance matrices and a confusion metric to assess the performance of our classifier on a testing set below.

We next set out to compile a list of amyloidosis-related proteins as input variables for a machine learning approach to achieve an even more objective proteome-based amyloid typing. This list was primarily informed by previous publications of Buxbaum *et al.* (3), Gottwald & Röcken (2) and Dogan *et al.* (22) and expanded by proteins identified in this study (e.g. variable immunoglobulin chains). The complete list of all amyloidosis-relevant proteins is provided in Supplemental Table S3. As a plausibility check we conducted a STRING network analysis of all selected amyloid-related proteins (Fig. 6*A*, left panel) resulting in a dense and well-interconnected network of those proteins matching with the STRING database. Moreover, enrichment analyses (Reactome and DisGeNet) yielding “Amyloid fiber formation”, “Plaque Amyloid”, and “primary systemic amyloidosis” as highest-ranking enrichment terms (*p* adjust < 1 × 10^−12^), further supporting the validity of the curated protein list (Fig. 6*A*, right panel)

The aforementioned protein list (n = 167) was then utilized as input variables for a supervised machine learning algorithm (XGBoost). The XGBoost was trained on a random split of 30 samples of all amyloidosis types with three or more cases per group (AA, ACal, ALκ, ALλ, ApoAIV, ApoAI, ATTR). The resulting classifications and associated probabilities for each sample in the testing set are illustrated in Figure 6*B*. As indicated by the general performance metrics and confusion matrix used to evaluate the classifier, 17 out of 18 samples in the testing set (94.4%) were correctly classified. Notably, the single misclassified sample (classified as ApoAIV instead of ALλ) had a probability value for ApoAIV of less than 0.5, whereas the vast majority of correctly classified samples demonstrated probability values exceeding 0.5. Additionally, the second most likely classification for the misclassified sample was, in fact, ALλ, matching with the correct diagnosis.

The high accuracy of the XGBoost classifier achieved despite the relatively small sample size (by machine learning standards), underscores the effectiveness of this approach. It further highlights the potential of integrating proteomic data with advanced machine learning techniques as a future direction for unbiased diagnostic amyloid typing.

## Discussion

Our study demonstrates the robustness and versatility of a comprehensive LC-MS/MS workflow with iBAQ APCS internal normalization as the key element for tissue-based amyloidosis typing. While various peptide identification workflows have been described previously (9, 16, 26, 28), an internal normalization approach utilizing iBAQ intensities and APCS as a reference protein has, to the best of our knowledge, not been published before. APCS appears an ideal housekeeping protein for normalization, as it represents one of the most well-studied amyloid signature proteins recognized by the ISA and is a crucial component of amyloid fibrils (3). APCS is supposed to be universally present in all the amyloid deposits constituting 10 to 15% of their total dry mass (18, 29, 30). Moreover, Palstrøm *et al.* developed an AI-based classification model for differentiating between amyloid-containing tissue samples and non-amyloidosis samples based on MS data using several combinations of amyloid signature proteins. In their validation cohort of 103 samples, APCS alone correctly classified 100% of the amyloid-containing samples (31). These findings provide strong justification for using APCS as an internal reference for normalization.

In former landmark publications as those from the Mayo Clinic (8, 26, 32), normalization of MS spectral counts (SC) per case was performed to address inter-sample variability. Although there is a correlation between protein abundance and SC, the use of high-resolution instruments and advanced algorithms renders intensity-based quantification techniques, such as iBAQ, a more precise and reliable choice (33). Since SC-based methods tend to overestimate the levels of outlier proteins (very high- and low-abundant proteins), they typically underperform compared to other quantification methods. iBAQ values offer better precision and accuracy and provide a higher correlation to known absolute protein quantities over a dynamic range spanning at least four orders of magnitude. Consequently, iBAQ values are frequently used to quantify differences between protein abundances within an individual sample, particularly in situations where replicates are not available (19, 34, 35, 36, 37). While this contrasts with the widespread use of LFQs in proteomic research, being particularly advantageous for inter-sample comparisons, it corresponds well to the demands of daily routine diagnostics. In this context, tissue samples are analyzed individually, and in cases such as endomyocardial biopsies, the limited quantity of available tissue often makes replicates impractical.

Enrichment procedures for amyloid-containing areas during tissue preparation, either by macrodissection (for relatively large and well-circumscribed amyloid deposits (e.g. cutaneous nodular amyloidosis)) or for smaller areas by LMD should always be prioritized, if possible. However, diffuse distribution of amyloid and low amounts of tissue, as frequently encountered in endomyocardial biopsies, often preclude these methods, requiring an alternative strategy such as using whole-dissected samples. The use of whole-dissected myocardial samples is supported by a recent elegant study by Noborn *et al.* (17). In this study, whole-dissected cardiac amyloidoses were successfully typed as ALκ, ALλ, ATTR and AA using mass spectrometry-based proteomic analysis on fresh frozen and FFPE tissue samples. However, Noborn *et al.* exclusively focused on the four most common heart-related amyloid types by analyzing the following proteins: Light chain (LC) λ (P0DOY2), LC κ (P01834), TTR (P02766) and SAA-1 (P0DJI8). With this approach 87% of amyloid cases were accurately typed achieving a 94% concordance rate with the original diagnosis. In contrast, our study also included rare and complex amyloid (sub)types and a more comprehensive list of amyloidosis-related proteins (Fig. 6*A* and Supplemental Table S3). This broader approach appears to be particularly relevant for two main reasons. First, cases such as ApoAIV and ApoAI amyloidosis would have been entirely overlooked without their inclusion. Secondly, at least in our cohort 30% of ALκ and 80% of ALλ cases did not produce the highest and thus type-defining peak for the proteins P01834 and P0DOY2, respectively, but a variable immunoglobulin light chain instead. Moreover, relying solely on these constant immunoglobulin regions would have resulted in missing 10% of ALκ and 60% of ALλ cases in our cohort due to very low intensities of the aforementioned proteins (Supplemental Table S5). These findings underscore the critical importance of adopting an expanded proteomic approach to ensure accurate amyloidosis typing, especially for rarer and more complex subtypes.

A key limitation of widely used proteomics data analysis workflows lies in the constraints of reference protein databases, such as SwissProt/UniProt, employed for database searches by protein identification algorithms. While MS/MS can detect peptides representing specific proteins or their variants, their spectra cannot be identified if the protein is absent from the reference database. This represents a significant challenge for proteomic subtyping of ATTR and AL amyloidosis, as many causative proteins may have variant sequences due to inherited or acquired mutations. This challenge can be partially addressed by creating a custom database containing all known hereditary and acquired amyloidogenic mutations before performing standard database searches or by employing sequence-tagging strategies designed to identify unexpected mutations (22). However, the availability of well-curated variant sequence databases remains limited, as SwissProt lacks comprehensive coverage. One alternative is to incorporate relevant protein sequences from the NCBI non-redundant database, although this database is less curated and contains redundant entries compared to SwissProt. To overcome this limitation, we utilized *de novo* sequencing from our MS/MS data to predict peptides. By searching these peptides in the NCBI database, we identified relevant proteins absent from SwissProt, significantly expanding the repertoire of amyloid protein variants available for analysis.

As already pointed out by Palstrøm *et al.*, interpretation of MS data remains somewhat subjective. To address this, they developed a support vector machine (SVM)-based approach to eliminate the need for manual expert interpretation. Using proteomics data from 75 laser-dissected CR-positive amyloid-containing biopsies and 78 negative biopsies, the Boruta method with a random forest classifier was employed to identify novel “amyloid signature proteins”. Notably, training the SVM algorithm with a combination of ApoA4, ApoE, and APCS—or APCS alone (as already pointed out above)—resulted in a perfect accuracy of 1.0 in distinguishing controls from amyloid-containing biopsies. Furthermore, the technique successfully classified individuals by the four most common amyloidosis subtypes in 102 out of 103 blinded cases (31). For this study, we evaluated three machine learning models—Random Forest (RF), SVM, and XGBoost—for their ability to accurately classify amyloidosis samples into the four most common subtypes (AA, ALκ, ALλ, and ATTR), as well as ACal, ApoAI, and ApoAIV amyloidoses. Among these, XGBoost demonstrated the best performance, achieving sensitivity, specificity, and accuracy of 0.96, 0.99, and 0.94, respectively. In comparison, the SVM model yielded lower performance, with respective sensitivity, specificity, and accuracy of 0.75, 0.96, and 0.78, while the RF model achieved 0.82, 0.98, and 0.89. XGBoost has been applied in various diagnostic contexts before, including identifying key biomarkers for early-stage heart failure (38) and predicting myocardial infarction (39). We anticipate that AI-based classification models will play a pivotal role in achieving more objective amyloid diagnostics in the future. However, this will require further validation with larger cohorts and the inclusion of all known amyloidosis types.

In Germany and many other European countries, proteomics remains underutilized in pathology institutes for amyloid typing, despite its recognized advantages and endorsements from professional societies (2, 3, 4, 6, 11, 12, 13, 14, 15). Several factors may contribute to this underuse: 1) IHC-based typing has a long-standing tradition, offering faster and relatively cost-effective results compared to LC-MS/MS; 2) in cases with very low amounts of amyloid deposits, IHC may demonstrate higher sensitivity 3) IHC stainings have established billing codes, while proteomic analyses so far lack such standardized reimbursements options; 4) historical concerns regarding the reliability and maturity of proteomic technologies have led to hesitancy in adoption despite significant advancements in recent years (40); 5) access to LC-MS/MS-based proteomics is typically limited to university hospitals and specialized centers, making it less accessible for routine use in many smaller pathology laboratories. Referring to the longstanding tradition of IHC, we believe that IHC-based amyloid typing is still a valid and effective diagnostic tool as long as it is performed by a very experienced pathologist using firmly established antibodies and who is well-versed in its nuances and limitations. This experience was ensured for this study (particularly for cardiac cases by K.K.), underlined by the fact that 100% of unequivocal IHC-typed cases could be confirmed by LC-MS/MS. Furthermore, in cases where IHC presented diagnostic challenges, there was the awareness of employing proteomics (once established on site) for achieving diagnostic clarification. We believe that concerns about the reliability of MS-based amyloidosis typing in general should be significantly alleviated by the MS studies referenced above (among others) and by this study with its side-by-side comparison of different analytical platforms reaching remarkably consistent results. The depth of proteomic analysis can vary depending on the LC-MS/MS platform, since analytical performance is influenced by factors such as the mass analyzers and column selection (41). Therefore, we were pleased to observe that our method performed reliably across various commonly used LC-MS/MS instrumentation combinations. Importantly, the side-by-side comparison also suggests that tissue-based amyloid diagnostics does not necessarily require the latest and most expensive instrumentation, potentially encouraging broader application of this technique beyond particularly privileged facilities and institutions.

## Data Availability

The mass spectrometry proteomics datasets generated during the current study are available in the ProteomeXchangeConsortium (http://proteomecentral.proteomexchange.org; ID: PXD061551) and MassIVE repository (https://massive.ucsd.edu/ProteoSAFe/static/massive.jsp; ID: MSV000097248).

## Supplemental Data

This article contains supplemental data.

## Conflict of Interest

The authors declare that they do not have any conflicts of interest with the content of this article.
