# Supplementary material for: Accurate MS-Based Diagnostic Amyloid Typing Using Endogenously Normalized Protein Intensities in Formalin-Fixed Paraffin-Embedded Tissue
Source: Mol Cell Proteomics. 2025 Jul 21;24(8):101040. doi: 10.1016/j.mcpro.2025.101040 (PMC12396419; doi:10.1016/j.mcpro.2025.101040)
Supplement: Supplementary Data [file mmc1.pdf]

Supplemental Data

Accurate MS-based diagnostic amyloid typing using endogenously normalized protein intensities in formalin-fixed paraffin-embedded tissue

Vanessa Hollfoth[1], Arslan Ali[1], Eyyub Bag[1], Philip Riemenschneider[1,2], Sven Mattern[1], Julia Luibrand[1], Mohamed Ali Jarboui[3], Kerstin Singer[1], Benjamin Goeppert[4], Mirita Franz-Wachtel[5], Martina Sauter[1], Shabnam Asadikomeleh[6], Tobias Feilen[7], Christian Hentschker[8], Silvia Ribback[6], Elke Hammer[8,9], Karsten Boldt[3,10], Frank Dombrowski[6], Oliver Schilling[7], Boris Macek[5], Marius Ueffing[3,10], Karin Klingel[1], Stephan Singer[1§]

Content **Page**

Suppl. Figure S1.	Grouping of similar proteins indicating the same amyloidosis type	2
Suppl. Figure S2.	Amyloidosis samples showing two or more highly abundant proteins	3
Suppl. Figure S3.	*De novo* sequencing results	4
Suppl. Figure S4.	Annotated spectra of the unique peptides used for the identification of type-defining amyloid protein.	5-6
Suppl. Table S2.	LC-MS/MS parameter (quantitative data)	7-12
Suppl. Table S3.	List of amyloidosis-relevant proteins	13-16
Suppl. Table S4.	QuPath results	17
Suppl. Table S5.	Amyloid typing based on constant immunoglobulin regions	18

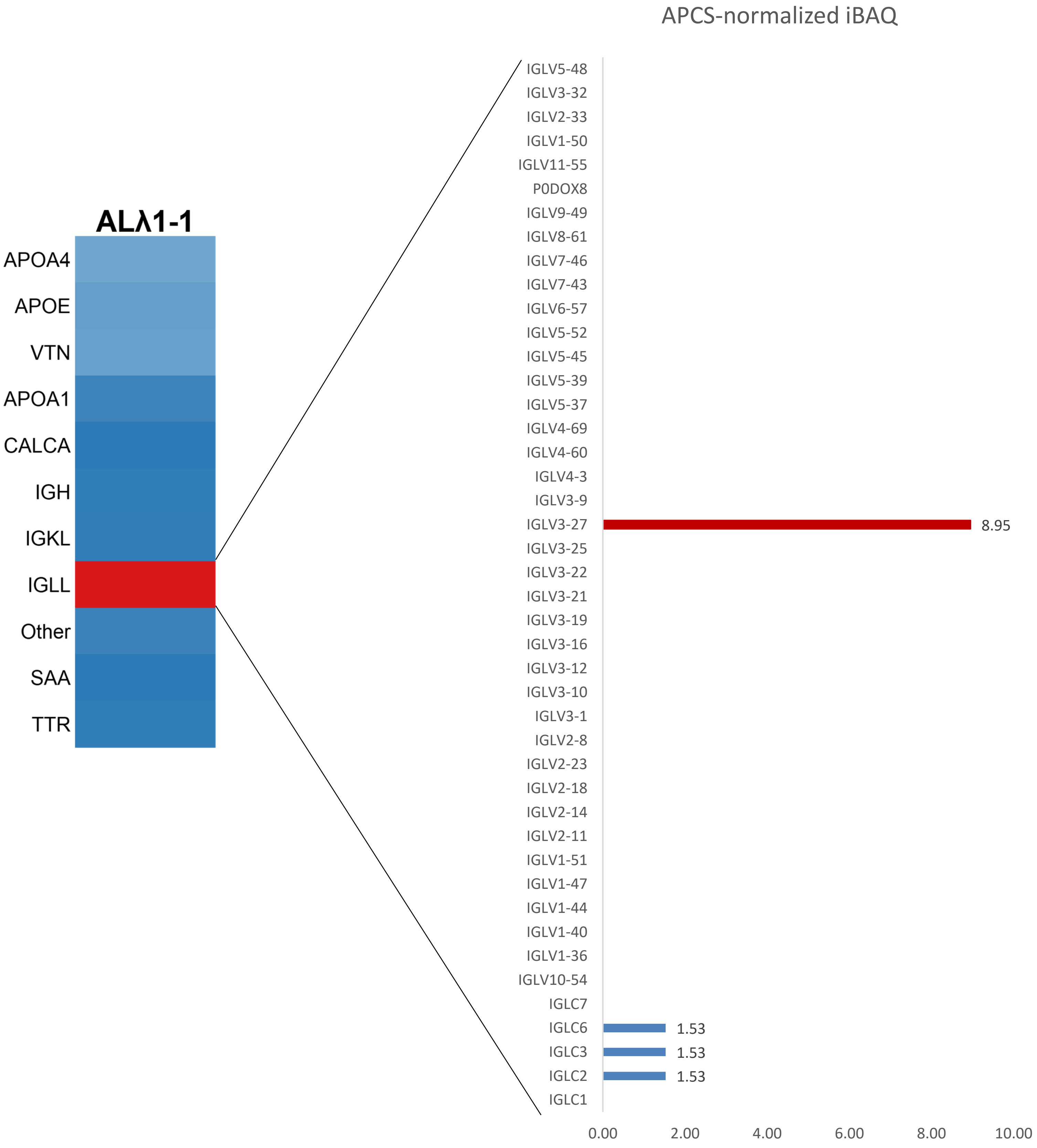

Suppl. Figure S1. Grouping of similar proteins indicating the same amyloidosis type

For proteins or protein groups that are part of immunoglobulin heavy chains, or kappa and lambda light chains, all identified constant and variable regions of each type are summarized as IGH, IGKL and IGLL, respectively. The heatmap (left) displays the maximal intensity measured in each group.

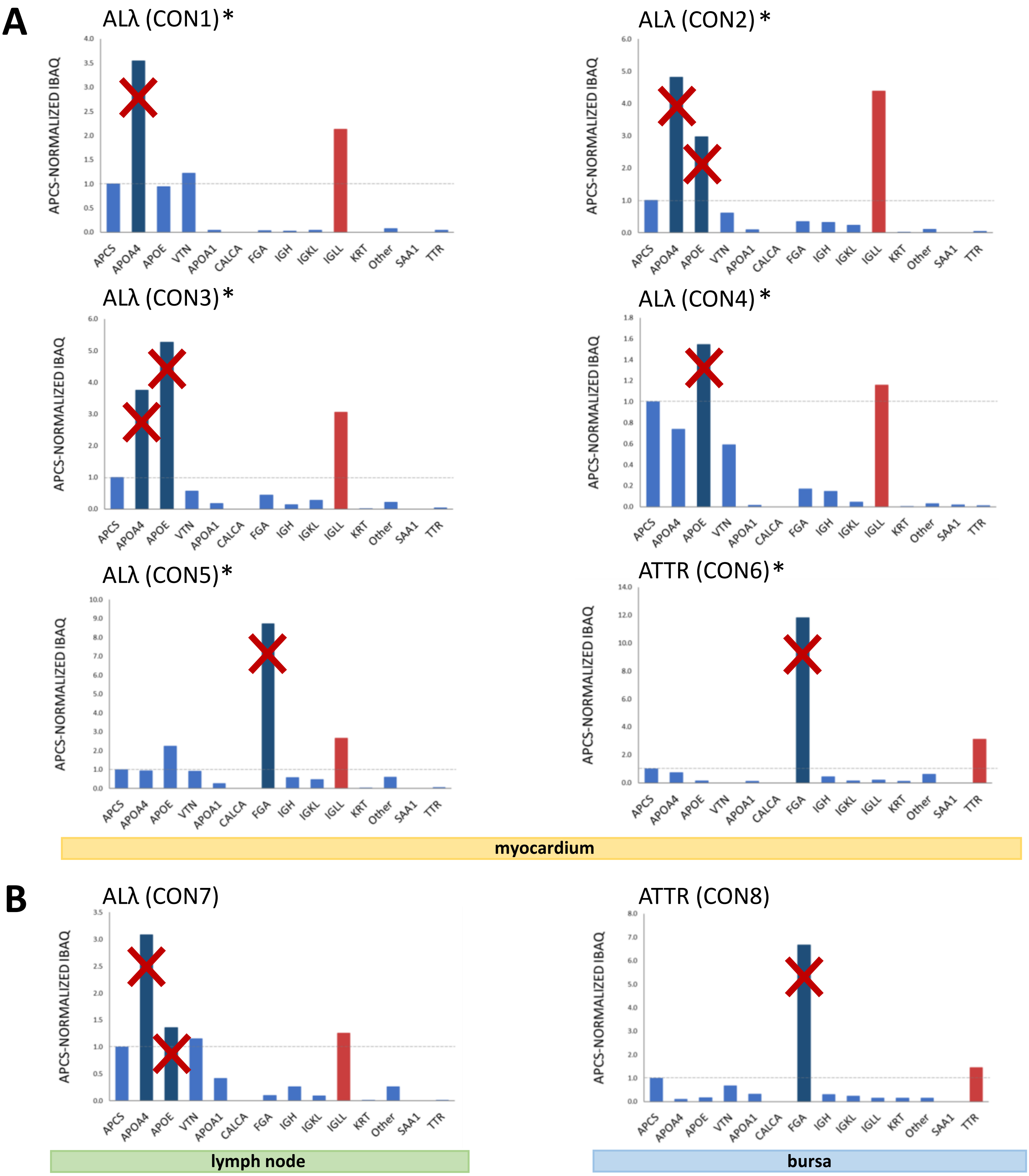

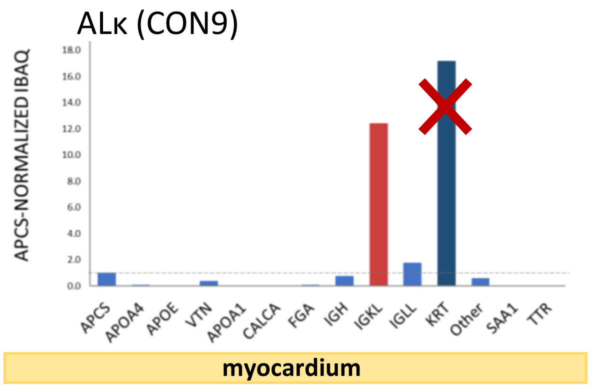

Suppl. Figure S2. Amyloidosis samples showing two or more highly abundant proteins

Amyloid typing of samples displaying high amounts of APOE, APOAIV, FGA or KRT, but without defining the amyloid type based on IHC and clinical context.

A

Sample	RT (min)	mz observed	z	pepMass	ppm error	Novor score	peptide	Novor aaScore
Unclear case #1	30.17	489.2875	2	976.5593	1.1	96.9	LLLYNVDK	99-99-99-99-97-96-96-92
Unclear case #2	39.96	781.8987	2	1561.781	1.1	84.2	GPQSPVLVMYQDTK	8-29-54-93-98-91-98-98-98-98-94-95-96-91

B

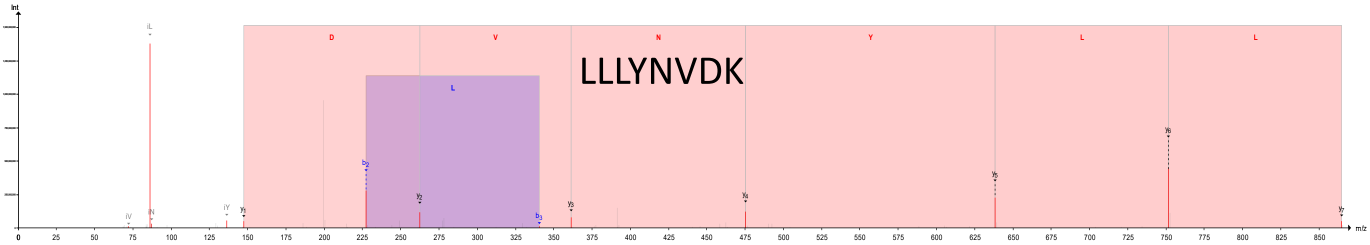

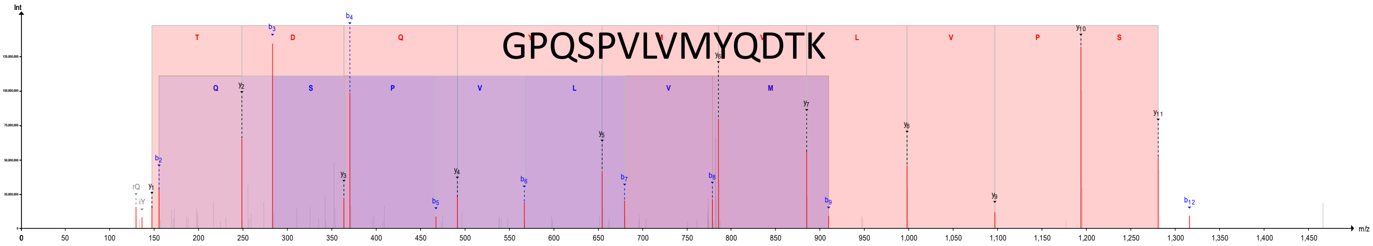

C

Sample	Description	Species	Max Score	Total Score	Query Cover.	E value	Per. ident	Acc. Len	Accession
Unclear case #1	immunoglobulin lambda light chain variable region	Homo sapiens	24	24	100%	7.5	75	110	CAI99684.1
Unclear case #2	immunoglobulin light chain variable region	Homo sapiens	43.1	43.1	85%	4.00E-06	100	106	UQZ09496.1

Suppl. Figure S3. *De novo* sequencing results

A) *De novo* sequencing results show peptides with good Novor score. B) MS/MS spectra annotation of peptide sequencing of the peptides. C) Top blast search results of the peptides search in NCBI human non-redundant database.

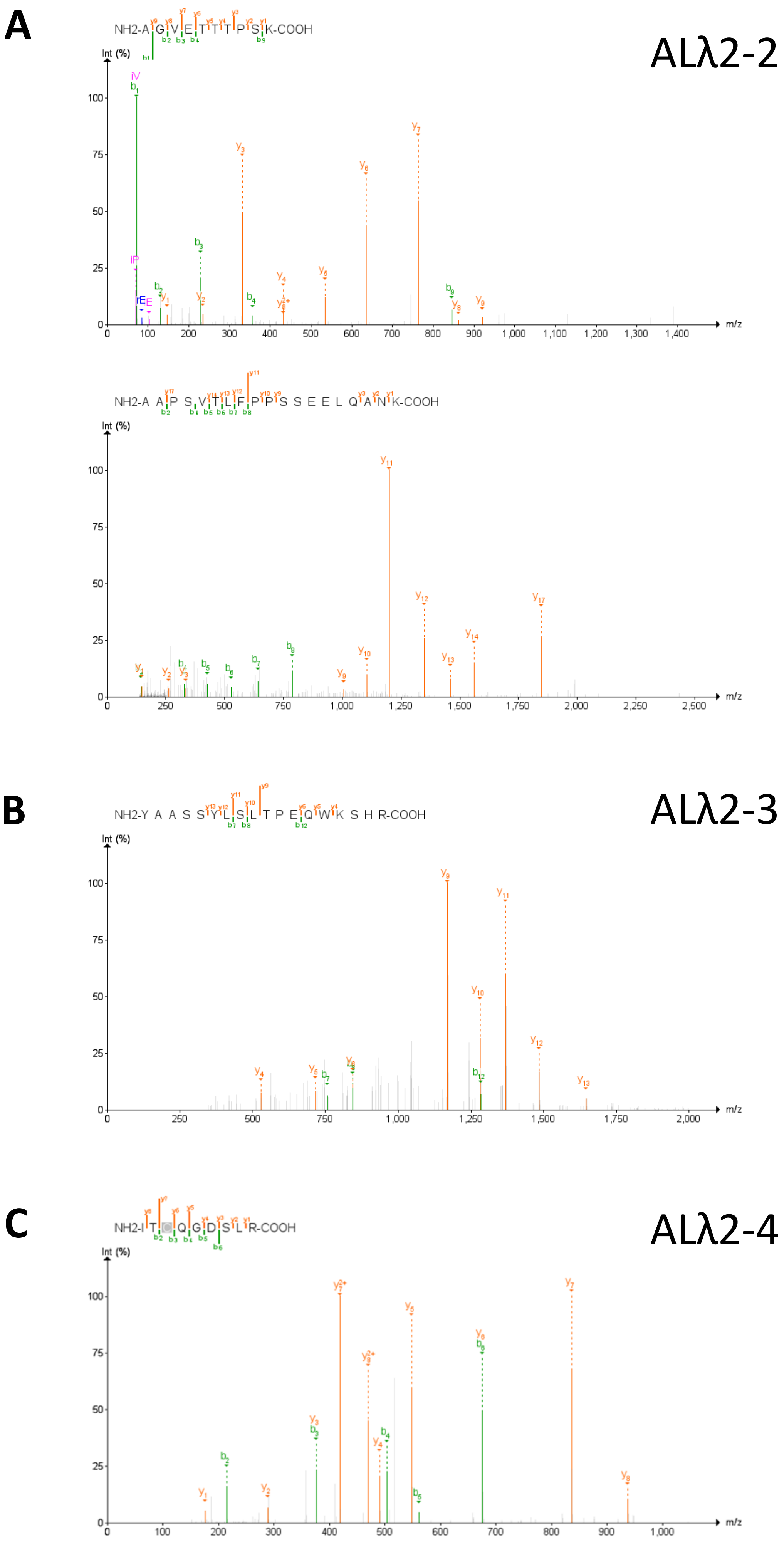

A
ALλ2-2
NH2-A G V E T T T P S K-COOH
Int (%)
m/z
NH2-A A P S V T L F P P S S E E L Q A N K-COOH
Int (%)
m/z
B
ALλ2-3
NH2-Y A A S S Y L S L T P E Q W K S H R-COOH
Int (%)
m/z
C
ALλ2-4
NH2-I T C Q G D S L R-COOH
Int (%)
m/z

D

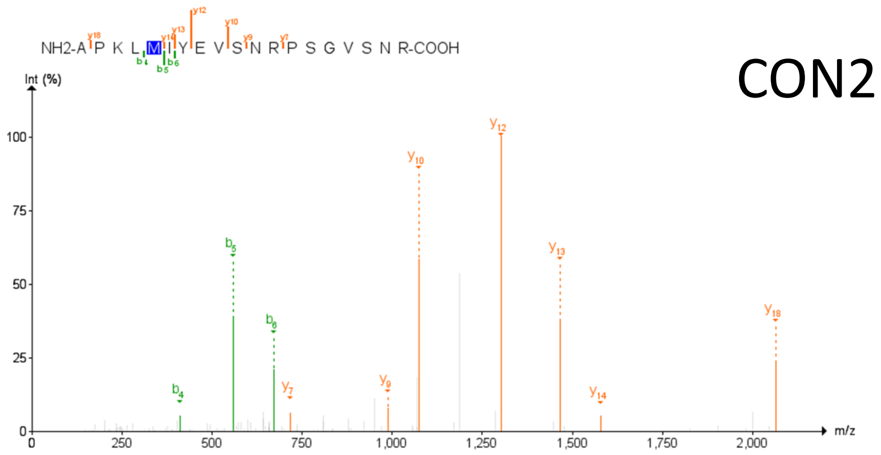

E

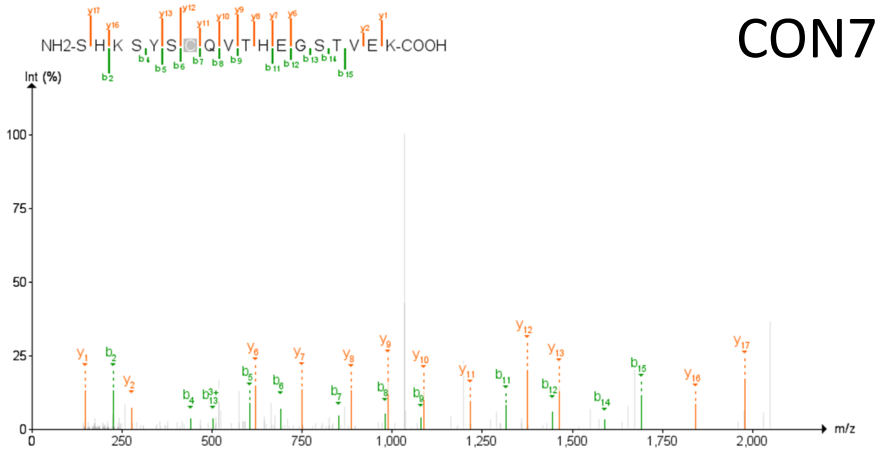

F

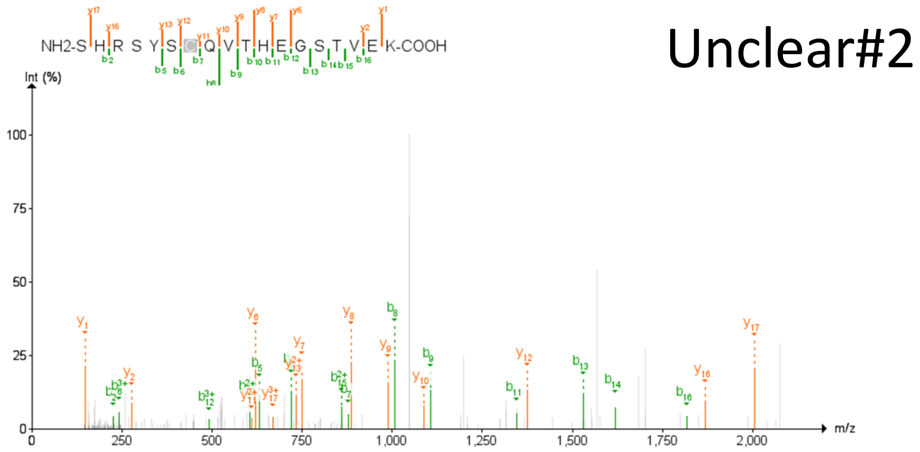

Suppl. Figure S4. Annotated spectra of the unique peptides used for the identification of type-defining amyloid protein

Annotated spectra representing the peptide-spectrum matches of the unique peptides for the amyloidosis type-defining proteins identified with fewer than two unique peptides. Visualization is generated using the PDV data visualization tool (https://github.com/wenbostar/PDV) .

Suppl. Table S2. LC-MS/MS parameter (quantitative data)

Q Exactive plus – CFMP TÜ

reversed phase liquid chromatography	**Ultimate 3000 RSLC (Thermo Scientific)**
Trap column	1 cm µPAC trapping column, monolithic silica chip with C18 chemistry: pillar diameter 5 µm, inter pillar distance 2.5 µm, pillar length/bed depth 18 µm, bed channel width of 2mm and a bed length of 10mm, porous shell thickness of 300nm and pore sizes between 100 to 200 Å (Thermo Scientific)
Analytical column	50 cm µPAC nano LC column, monolithic silica chip with C18 chemistry, pillar diameter 5 µm, inter pillar distance 2.5 µm, pillar length/bed depth 18 µm, bed channel width of 315 µm and a bed length of 50 cm porous shell thickness of 300nm and pore sizes between 100 to 200 Å (Thermo Scientific)
Flow rate	300 nl/min
column oven temperature	40°C
buffer system	binary buffer system consisting of 2% ACN/0.1% FA (buffer A) and 80% ACN/0.08% FA (buffer B)
gradient	gradient of buffer B: 3 min 2%, 39 min 2% to 30%, 1 min 30% to 95%, 6 min 95%, 1 min 95% to 2%, 20 min 2%
Mass spectrometer	Q Exactive Plus mass spectrometer (Thermo Scientific)
operation mode	data-dependent acquisition (DDA)
electrospray	Nanospray Flex Ion Source
Full MS	
MS scan resolution	70,000
Full MS AGC target	3.00E+06
maximum ion injection time for the MS scan	100 ms
Scan range	335 to 1500 *m/z*
Spectra data type	Profile
dd-MS2	
Resolution	17,500
MS/MS AGC target	1.00E+05
maximum ion injection time for the MS/MS scans	200 ms
Spectra data type	centroid
selection for MS/MS	Top 10
isolation window	2 m/z
Scan range	200-2000 m/z
Intensity threshold	5.0E+04
Monoisotopic precursor selection rejected	Unassigned, +1 charges
Dynamic exclusion	20 sec
Fixed first mass	200
dissociation mode	higher energy collisional dissociation (HCD)
normalized collision energy	fixed, 26%

Orbitrap Fusion – CFMP TÜ

reversed phase liquid chromatography	**Ultimate 3000 RSLC (Thermo Scientific)**
Trap column	µPAC Trapping Column (PharmaFluidics), 2 mm bed length, pillar diameter 5 µm, inter-pillar distance 2.5 µm, bed depth 18 µm, external porosity 9%
Analytical column	50 cm µPAC nano-LC column (PharmaFluidics), pillar diameter 5 µm, inter-pillar distance 2.5 µm, bed depth 18 µm, external porosity 59%
Flow rate	300 nl/min
column oven temperature	40°C
buffer system	binary buffer system consisting of 2% ACN/0.1% FA (buffer A) and 80% ACN/0.08% FA (buffer B)
gradient	gradient of buffer B: 3 min 2%, 82 min 2% to 30%, 5 min 30% to 95%, 5 min 95%, 5 min 95% to 2%, 20 min 2%
Valve switching events	3 min: direct sample flow onto analytical column, 115 min: return valve for flushing/re-equilibration
Pump max. pressure	250 bar
Flow ramp rates	0.3 µL/min² (up/down) for the nano–LC pump
Mass spectrometer	Orbitrap Fusion mass spectrometer (Thermo Scientific)
operation mode	data-dependent acquisition (DDA)
Full MS	
MS scan resolution	120,000
Full MS AGC target	4.00E+05
maximum ion injection time	50 ms
Scan range	335 to 1500 *m/z*
Spectra data type	Profile
RF Lens (%)	60
dd-MS2	
Data dependent mode	Cycle Time
MSn Level	2 (ddMSnScan)
MS/MS AGC target	2.00E+03
maximum ion injection time	300 ms
Spectra data type	centroid
Detector type	Ion Trap (Rapid scan)
isolation window	1.6 m/z
Scan range	Automatic
Intensity threshold	5.0E+03
Monoisotopic precursor selection rejected	Peptide (“Relax restrictions if too few precursors”)
Charge state filter	Include z = 2–7; exclude undetermined charge states
Dynamic exclusion	Repeat count = 1, exclusion = 60 s, mass tolerance ±10 ppm, exclude isotopes = True
dissociation mode	higher energy collisional dissociation (HCD)
normalized collision energy	fixed, 30%

Q Exactive HF – PCT TÜ	
reversed phase liquid chromatography	**Easy nLC 1200 UHPLC (Thermo Scientific)**
Trap column	-
Analytical column	75 µm ID PicoTip fused silica emitter (New Objective) in-house packed with ReproSil-Pur C18-AQ 1.9 µm resin (Dr Maisch HPLC GmbH)
Flow rate	200 nl/min
column oven temperature	40°C
buffer system	binary buffer system consisting of 0.1% formic acid in HPLC-grade water (solvent A) and 80% ACN in 0.1% formic acid (solvent B)
gradient	**ALλ2-1** (**500ng injected**; **60min gradient**):<br>10% to 33% B in 43min, 33% to 50% B in 3min at a flow rate of 200nl/min 50% to 90% B in 3min, 90%B to 50% B for 8min at a flow rate of 500nl/min<br><br>**ATTR-R3** (**250ng** injected; **90min gradient**):<br>10% to 33% B in 73min, 33% to 50% B in 3min at a flow rate of 200nl/min 50% to 90% B in 3min, 90%B to 50% B for 8min at a flow rate of 500nl/min
Mass spectrometer	**Q Exactive HF mass spectrometer (Thermo Scientific)**
operation mode	data-dependent acquisition (DDA)
electrospray	Nanospray Flex Ion Source
Full MS	
MS scan resolution	60,000
Full MS AGC target	3.00E+06
maximum ion injection time for the MS scan	25 ms
Scan range	300 to 1650 *m/z*
Spectra data type	profile
dd-MS2	
Resolution	30,000
MS/MS AGC target	1.00E+05
maximum ion injection time for the MS/MS scans	45 ms
Spectra data type	profile
selection for MS/MS	Top 12
isolation window	1.4 m/z
Scan range	200-2000 m/z
Intensity threshold	1E+05
Monoisotopic precursor selection rejected	+1 and (+6) – (+8), and > (+8) charged ions
Dynamic exclusion	30 sec
Fixed first mass	100
dissociation mode	higher energy collisional dissociation (HCD)
normalized collision energy	fixed, 27%

Q Exactive plus – HGW	
reversed phase liquid chromatography	**Ultimate 3000 RSLC (Thermo Scientific)**
Trap column	75 µm inner diameter, packed with 3 µm C18 particles (Acclaim PepMap100, Thermo Scientific)
Analytical column	75 µm inner diameter, packed with 2.6 µm C18 particles (Accucore, 25 cm, Thermo Scientific)
Flow rate	300 nl/min
column oven temperature	40°C
buffer system	binary buffer system consisting of 0.1% acetic acid in HPLC-grade water (buffer A) and 100% ACN in 0.1% acetic acid (buffer B)
gradient	gradient of buffer B: 2min 2% to 5 %, 8min 5%, 120min 5% to 25%, 5min 25 to 40%, 2 min 40% to 90%, 5 min 90%, 1 min 90% to 2%, 7 min 2%
Mass spectrometer	Q Exactive Plus mass spectrometer (Thermo Scientific)
operation mode	data-dependent acquisition (DDA)
electrospray	Nanospray Flex Ion Source
Full MS	
MS scan resolution	70,000
Full MS AGC target	3.00E+06
maximum ion injection time for the MS scan	120 ms
Scan range	350 to 1650 *m/z*
Spectra data type	profile
dd-MS2	
Resolution	17,500
MS/MS AGC target	2.00E+05
maximum ion injection time for the MS/MS scans	120 ms
Spectra data type	centroid
selection for MS/MS	Top 10
isolation window	3 m/z
Scan range	200-2000 m/z
Intensity threshold	8.3E+04
Monoisotopic precursor selection rejected	+1 and +7, +8, and >+8 charged ions
Dynamic exclusion	30 sec
Fixed first mass	200
dissociation mode	higher energy collisional dissociation (HCD)
normalized collision energy	fixed, 27.5%

LTQ Orbitrap Velos – HGW	
reversed phase liquid chromatography	**nano-Acquity UPLC (Waters Corp)**
trap column	nanoACQUITY UPLC Symmetry C18 Trap Column, 100Å, 5 µm, 180 µm x 20 mm, 2G, V/M, Waters
analytical column	BEH C18 nanoACQUITY Column 10K psi, 130Å, 1.7 µm, 100 µm X 100 mm, Waters
flow rate	400 nl/min
column oven temperature	40°C
buffer system	binary buffer system consisting of 0.1% acetic acid in HPLC-grade water (buffer A) and 100% ACN in 0.1% acetic acid (buffer B)
gradient	gradient of buffer B: 5min 1% to 5 %, 63min 5% to 25%, 25min 25 to 60%, 2min 60% to 99%, 2min 99%, 1min 99% to 1%, 5 min 1%
Mass spectrometer	**LTQ Orbitrap Velos**
operation mode	data-dependent acquisition (DDA)
electrospray	Nanospray Flex Ion Source
Full MS	
MS scan resolution	30,000
AGC target	1e6
maximum ion injection time for the MS scan	10 ms
scan range	325 to 1525 *m/z*
spectra data type	profile
dd-MS2	
resolution	7,500
MS/MS AGC target	3e4
minimum ACG target	-
intensity threshold	2e3
maximum ion injection time for the MS/MS scans	100 ms
number of MS/MS scans	Top 20
spectra data type	centroid
selection for MS/MS	1
isolation window	2.0 Da
fixed first mass	-
dissociation mode	collisional induced dissociation (CID)
normalized collision energy	35
charge exclusion	unassigned, 1, >3
dynamic exclusion	60 sec

TimsTOF Flex – FR		
reversed phase liquid chromatography	**Evosep One (Evosep)**	
Trap column	*not used*	
Analytical column	EV1137 performance 15 cm reversed phase column (Evosep)	
Flow rate	500 nl/min	
column oven temperature	40°C	
buffer system	binary buffer system consisting of 0.1% formic acid in HPLC-grade water (buffer A) and 100% ACN in 0.1% formic acid (buffer B)	
gradient	Evosep 30 Samples-per-day method, 44 min gradient	
Mass spectrometer	TimsTOF Flex Mass Spectrometer (Bruker Daltonics)	
operation mode	data-dependent acquisition with parallel accumulation serial fragmentation (DDA-PASEF)	
electrospray	CaptiveSpray	
MS settings		
Scan Begin	100 m/z	
Scan End	1700 m/z	
Ion Polarity	positive	
Scan mode	PASEF	
TIMS settings		
Mode	Custom	
1/K0 Start	0.6 $V*s/cm^2$	
1/K0 End	1.6 $V*s/cm^2$	
Ramp Time	100 ms	
Accu. Time	100 ms	
Duty Cycle	100 %	
Ramp Rate	9.43 Hz	
Precursor Ions		
Number of PASEF ramps	10	
Total Cycle Time	1.17 s	
Charge Minimum	0	
Charge Maximum	5	
MS repetitions	1x	
Cycle Overlap	4	
Summation Width	25 pts.	
Max. No. of Peaks	3	
Active Exclusion		
Release after	0.40 min	
Collision Energy Settings	**1/K0 [$V*s/cm^2$]**	**Collision Energy [eV]**
	0.60	20.00
	1.60	59.00
Isolation Width Settings	**Mass [m/z]**	**Width [m/z]**
	700	2.00
	800	3.00

Suppl. Table S3. List of amyloidosis-relevant proteins

UniProt ID	Gen Name	Protein Name	Group
P02743	APCS	Serum amyloid P-component	APCS
P06727	APOA4	Apolipoprotein A-IV	APOA4
P02649	APOE	Apolipoprotein E	APOE
P04004	VTN	Vitronectin	VTN
P37840	SNCA	alpha-Synuclein	Other
P02647	APOA1	Apolipoprotein A-I	APOA1
P02652	APOA2	Apolipoprotein A-II	Other
P04114	APOB	Apolipoprotein B-100; Apolipoprotein B-48	Other
P02654	APOC1	Apolipoprotein C-I	Other
P02655	APOC2	Apolipoprotein C-II	Other
P02656	APOC3	Apolipoprotein C-III	Other
Q13790	APOF	Apolipoprotein F	Other
Q9BQE5	APOL2	Apolipoprotein L2	Other
O95445	APOM	Apolipoprotein M	Other
P61769	B2M	Beta-2-microglobulin	Other
P05067	APP	beta-amyloid Protein	Other
P01258	CALCA	Calcitonin	CALCA
P43235	CTSK	Cathepsin K	Other
P01024	C3	Complement C3	Other
P02748	C9	Complement component C9	Other
Q15517	CDSN	Corneodesmosin	Other
P01034	CST3	Cystatin C	Other
Q12805	EFEMP1	EGF-containing fibulin-like extracellular matrix protein 1	Other
P02671	FGA	Fibrinogen alpha chain	Other, FGA
P47929	LGALS7	Galectin-7	Other
P06396	GSN	Gelsolin	Other
P0DOX2	N/A	Immunoglobulin alpha-2 heavy chain	IGH
P0DOX3	N/A	Immunoglobulin delta heavy chain	IGH
P0DOX4	N/A	Immunoglobulin epsilon heavy chain	IGH
P0DOX5	N/A	Immunoglobulin gamma-1 heavy chain	IGH
P01876	IGHA1	Immunoglobulin heavy constant alpha 1	IGH
P01877	IGHA2	Immunoglobulin heavy constant alpha 2	IGH
P01880	IGHD	Immunoglobulin heavy constant delta	IGH
P01854	IGHE	Immunoglobulin heavy constant epsilon	IGH
P01857	IGHG1	Immunoglobulin heavy constant gamma 1	IGH
P01859	IGHG2	Immunoglobulin heavy constant gamma 2	IGH
P01860	IGHG3	Immunoglobulin heavy constant gamma 3	IGH
P01861	IGHG4	Immunoglobulin heavy constant gamma 4	IGH
P01871	IGHM	Immunoglobulin heavy constant mu	IGH
P0DOY5	IGHD1-1	Immunoglobulin heavy diversity 1-1	IGH
P01591	JCHAIN	Immunoglobulin J chain	IGH
P01834	IGKC	Immunoglobulin kappa constant	IGKL
P0DOX7	N/A	Immunoglobulin kappa light chain	IGKL
A0A0C4DH73	IGKV1-12	Immunoglobulin kappa variable 1-12	IGKL
P0DP09	IGKV1-13	Immunoglobulin kappa variable 1-13	IGKL

P04430	IGKV1-16	Immunoglobulin kappa variable 1-16	IGKL
P01599	IGKV1-17	Immunoglobulin kappa variable 1-17	IGKL
A0A075B6S5	IGKV1-27	Immunoglobulin kappa variable 1-27	IGKL
P01594	IGKV1-33	Immunoglobulin kappa variable 1-33	IGKL
P01597	IGKV1-39	Immunoglobulin kappa variable 1-39	IGKL
P01602	IGKV1-5	Immunoglobulin kappa variable 1-5	IGKL
A0A0C4DH72	IGKV1-6	Immunoglobulin kappa variable 1-6	IGKL
A0A0C4DH67	IGKV1-8	Immunoglobulin kappa variable 1-8	IGKL
A0A0C4DH69	IGKV1-9	Immunoglobulin kappa variable 1-9	IGKL
P01611	IGKV1D-12	Immunoglobulin kappa variable 1D-12	IGKL
A0A0B4J2D9	IGKV1D-13	Immunoglobulin kappa variable 1D-13	IGKL
P01601	IGKV1D-16	Immunoglobulin kappa variable 1D-16	IGKL
A0A075B6S4	IGKV1D-17	Immunoglobulin kappa variable 1D-17	IGKL
P01593	IGKV1D-33	Immunoglobulin kappa variable 1D-33	IGKL
P04432	IGKV1D-39	Immunoglobulin kappa variable 1D-39	IGKL
A0A0B4J1Z2	IGKV1D-43	Immunoglobulin kappa variable 1D-43	IGKL
A0A087WSZ0	IGKV1D-8	Immunoglobulin kappa variable 1D-8	IGKL
A0A0C4DH68	IGKV2-24	Immunoglobulin kappa variable 2-24	IGKL
A0A075B6P5	IGKV2-28	Immunoglobulin kappa variable 2-28	IGKL
A2NJV5	IGKV2-29	Immunoglobulin kappa variable 2-29	IGKL
P06310	IGKV2-30	Immunoglobulin kappa variable 2-30	IGKL
A0A087WW87	IGKV2-40	Immunoglobulin kappa variable 2-40	IGKL
A0A0A0MRZ7	IGKV2D-26	Immunoglobulin kappa variable 2D-26	IGKL
P01615	IGKV2D-28	Immunoglobulin kappa variable 2D-28	IGKL
A0A075B6S2	IGKV2D-29	Immunoglobulin kappa variable 2D-29	IGKL
A0A075B6S6	IGKV2D-30	Immunoglobulin kappa variable 2D-30	IGKL
P01614	IGKV2D-40	Immunoglobulin kappa variable 2D-40	IGKL
P04433	IGKV3-11	Immunoglobulin kappa variable 3-11	IGKL
P01624	IGKV3-15	Immunoglobulin kappa variable 3-15	IGKL
P01619	IGKV3-20	Immunoglobulin kappa variable 3-20	IGKL
A0A0A0MRZ8	IGKV3D-11	Immunoglobulin kappa variable 3D-11	IGKL
A0A087WSY6	IGKV3D-15	Immunoglobulin kappa variable 3D-15	IGKL
A0A0C4DH25	IGKV3D-20	Immunoglobulin kappa variable 3D-20	IGKL
A0A0C4DH55	IGKV3D-7	Immunoglobulin kappa variable 3D-7	IGKL
P06312	IGKV4-1	Immunoglobulin kappa variable 4-1	IGKL
P06315	IGKV5-2	Immunoglobulin kappa variable 5-2	IGKL
A0A0C4DH24	IGKV6-21	Immunoglobulin kappa variable 6-21	IGKL
A0A0A0MT36	IGKV6D-21	Immunoglobulin kappa variable 6D-21	IGKL
P0CG04	IGLC1	Immunoglobulin lambda constant 1	IGLL
P0DOY2	IGLC2	Immunoglobulin lambda constant 2	IGLL
P0DOY3	IGLC3	Immunoglobulin lambda constant 3	IGLL
P0CF74	IGLC6	Immunoglobulin lambda constant 6	IGLL
A0M8Q6	IGLC7	Immunoglobulin lambda constant 7	IGLL
A0A075B6I4	IGLV10-54	Immunoglobulin lambda variable 10-54	IGLL
A0A0B4J1U3	IGLV1-36	Immunoglobulin lambda variable 1-36	IGLL
P01703	IGLV1-40	Immunoglobulin lambda variable 1-40	IGLL
P01699	IGLV1-44	Immunoglobulin lambda variable 1-44	IGLL
P01700	IGLV1-47	Immunoglobulin lambda variable 1-47	IGLL
P01701	IGLV1-51	Immunoglobulin lambda variable 1-51	IGLL
P01706	IGLV2-11	Immunoglobulin lambda variable 2-11	IGLL

P01704	IGLV2-14	Immunoglobulin lambda variable 2-14	IGLL
A0A075B6J9	IGLV2-18	Immunoglobulin lambda variable 2-18	IGLL
P01705	IGLV2-23	Immunoglobulin lambda variable 2-23	IGLL
P01709	IGLV2-8	Immunoglobulin lambda variable 2-8	IGLL
P01715	IGLV3-1	Immunoglobulin lambda variable 3-1	IGLL
A0A075B6K4	IGLV3-10	Immunoglobulin lambda variable 3-10	IGLL
A0A075B6K2	IGLV3-12	Immunoglobulin lambda variable 3-12	IGLL
A0A075B6K0	IGLV3-16	Immunoglobulin lambda variable 3-16	IGLL
P01714	IGLV3-19	Immunoglobulin lambda variable 3-19	IGLL
P80748	IGLV3-21	Immunoglobulin lambda variable 3-21	IGLL
A0A075B6J6	IGLV3-22	Immunoglobulin lambda variable 3-22	IGLL
P01717	IGLV3-25	Immunoglobulin lambda variable 3-25	IGLL
P01718	IGLV3-27	Immunoglobulin lambda variable 3-27	IGLL
A0A075B6K5	IGLV3-9	Immunoglobulin lambda variable 3-9	IGLL
A0A075B6K6	IGLV4-3	Immunoglobulin lambda variable 4-3	IGLL
A0A075B6I1	IGLV4-60	Immunoglobulin lambda variable 4-60	IGLL
A0A075B6H9	IGLV4-69	Immunoglobulin lambda variable 4-69	IGLL
A0A075B6J1	IGLV5-37	Immunoglobulin lambda variable 5-37	IGLL
A0A0G2JS06	IGLV5-39	Immunoglobulin lambda variable 5-39	IGLL
A0A087WSX0	IGLV5-45	Immunoglobulin lambda variable 5-45	IGLL
A0A0A0MRZ9	IGLV5-52	Immunoglobulin lambda variable 5-52	IGLL
P01721	IGLV6-57	Immunoglobulin lambda variable 6-57	IGLL
P04211	IGLV7-43	Immunoglobulin lambda variable 7-43	IGLL
A0A075B6I9	IGLV7-46	Immunoglobulin lambda variable 7-46	IGLL
A0A075B6I0	IGLV8-61	Immunoglobulin lambda variable 8-61	IGLL
A0A0B4J1Y8	IGLV9-49	Immunoglobulin lambda variable 9-49	IGLL
P0DOX8	N/A	Immunoglobulin lambda-1 light chain	IGLL
P0DOX6	N/A	Immunoglobulin mu heavy chain	IGH
P01308	INS	Insulin	Other
Q9Y287	ITM2B	Integral membrane protein 2B (ITM2B)	Other
P10997	IAPP	Islet Amyloid Polypeptide/Amylin	Other
P02533	KRT14	Keratin, type I cytoskeletal 14	Other, KRT
P13647	KRT5	Keratin, type II cytoskeletal 5	Other, KRT
Q15582	TGFBI	Keratoepithelin	Other
Q08431	MFGE8	Lactadherin (Medin)	Other
P02788	LTF	Lactotransferrin /Lactoferrin	Other
O14960	LECT2	Leukocyte cell-derived chemotaxin-2 (LECT2)	Other
P11686	SFTPC	Lung Surfactant Protein C	Other
P61626	LYZ	Lysozyme C	Other
P01160	NPPA	Natriuretic peptides A	Other
A1E959	ODAM	Odontogenic ameloblast-associated protein	Other
Q99650	OSMR	Oncostatin-M specific receptor subunit beta/OSMR	Other
P04637	TP53	p53	Other
P01833	PIGR	Polymeric immunoglobulin receptor; Secretory component	Other
P04156	PRNP	Prion Protein	Other
A0A075B6H8	IGKV1D-42	Probable non-functional immunoglobulin kappa variable 1D-42	IGKL

A0A075B6R9	IGKV2D-24	Probable non-functional immunoglobulin kappa variable 2D-24	IGKL
A0A075B6H7	IGKV3-7	Probable non-functional immunoglobulin kappa variable 3-7	IGKL
A0A0C4DH26	IGKV6D-41	Probable non-functional immunoglobulin kappa variable 6D-41	IGKL
A0A075B6I3	IGLV11-55	Probable non-functional immunoglobulin lambda variable 11-55	IGLL
A0A075B6I6	IGLV1-50	Probable non-functional immunoglobulin lambda variable 1-50	IGLL
A0A075B6J2	IGLV2-33	Probable non-functional immunoglobulin lambda variable 2-33	IGLL
A0A0A0MS00	IGLV3-32	Probable non-functional immunoglobulin lambda variable 3-32	IGLL
A0A075B6I7	IGLV5-48	Probable non-functional immunoglobulin lambda variable 5-48	IGLL
A0A075B6S9	IGKV1-37	Probable non-functional immunoglobulinn kappa variable 1-37	IGKL
P0DSN7	IGKV1D-37	Probable non-functional immunoglobulinn kappa variable 1D-37	IGKL
P01236	PRL	Prolactin	Other
P04279	SEMG1	Semenogelin-1	Other
P0DJI8	SAA1	Serum amyloid A-1 protein	SAA
P0DJI9	SAA2	Serum amyloid A-2 protein	SAA
P35542	SAA4	Serum amyloid A-4 protein	SAA
P10636	MAPT	Tau	Other
P02766	TTR	Transthyretin	TTR
Q9NUM4	TMEM106B	Transmembrane proteine 106B	Other
P61278	SST	Somatostatin	Other
P01275	GCG	Pro-Glucagon	Other
P01270	PTH	Parathyroid hormone	Other
N/A	N/A	Enfurvitide	Other
P18510	IL1RN	Interleukin-1 receptor antagonist protein	Other
CAI99684.1	IGLL	immunoglobulin lambda light chain variable region, partial	IGLL
UQZ09496	IGLL	immunoglobulin light chain variable region	IGLL

Suppl. Table S4. QuPath results

ID	QuPath %	IHC	Diagnosis
**ATTR1-7**	59.5	ATTR	ATTR
**ATTR1-1**	40.6	ATTR	ATTR
**ATTR1-8**	40.1	ATTR	ATTR
**ALλ1-2**	34.1	AL lambda	AL lambda
**ATTR2-1**	28	ATTR/AL kappa	ATTR
**ALλ1-3**	27.5	AL lambda	AL lambda
**ALλ1-5**	26	AL lambda	AL lambda
**ATTR1-5**	24.4	ATTR	ATTR
**ATTR1-2**	22.2	ATTR	ATTR
**CON1**	19.6	AL lambda	AL lambda
**CON4**	16.7	AL lambda	AL lambda, also high ApoE and ApoAIV
**ApoAIV2-4**	16	AL lambda, kappa (slightly positive)	ApoAIV
**ATTR1-6**	14.3	ATTR	ATTR
**ALλ1-1**	9.8	AL lambda	AL lambda
**CON6**	8.9	AL lambda	AL lambda
**CON2**	7.9	AL lambda	AL lambda
**ALλ1-4**	7.6	AL lambda	AL lambda
**ATTR1-3**	7.4	ATTR	ATTR
**CON3**	7	AL lambda	AL lambda, high ApoE
**ATTR1-9**	6.2	ATTR	ATTR
**AH/AL2-1**	3.7	AL lambda	AH/AL lambda
**ATTR1-4**	2.3	ATTR	ATTR
**CON7**	1.7	ATTR	ATTR

different results in IHC and proteomic (highlighted rows: ATTR2-1, ApoAIV2-4, AH/AL2-1)

Suppl. Table S5. Amyloid typing based on constant immunoglobulin regions

ID	Type-defining protein	Ratio of type-defining protein/APCS	Ratio of P01834/APCS
**ALκ1-1**	P01834		
**ALκ1-2**	P01594	2.53	0.45
**ALκ2-1**	P01834		
**ALκ2-2**	P01624	23.09	20.86
**ALκ2-3**	P01834		
**ALκ2-4**	P01834		
**ALκ2-5**	P01834		
**ALκ2-6**	P01834		
**ALκ2-7**	P01624	6.11	1.47
**ALκ2-8**	P01834		
**ID**	**Type-defining protein**	**Ratio of type-defining protein/APCS**	**Ratio of P0D0Y2/APCS**
**ALλ1-1**	P80748	6.94	0.51
**ALλ1-2**	P01717	4.21	0.01
**ALλ1-3**	P0CG04	5.77	0
**ALλ1-4**	P01715	5.36	0.2
**ALλ1-5**	P01714	14.87	1.4
**ALλ1-6**	P0D0Y2		
**ALλ2-1**	P01701	13.48	3.43
**ALλ2-2**	P0D0Y2		
**ALλ2-3**	P0CG04	2.31	0.61
**ALλ2-4**	P01714	1.01	0.04

Standard proteins

**P01834**	**Ig kappa constant**
**P0D0Y2**	**Ig lambda constant 2**

(shaded)	**Standard protein is not the highest peak**
0	**Standard protein is lower than intensity of APCS (no amyloidosis typing possible)**
